# The Papua New Guinea snakebite partnership: a retrospective study of snakebite epidemiology and management, 2018-2023

**DOI:** 10.3389/ftox.2026.1961869

**Published:** 2026-10-12

**Authors:** Geno Roalakona, Andrew Maru, Timothy N. W. Jackson, Teatulohi Matainaho, Garry Nou, Nakapi Tefuarani, Andrew D. Watt

**Affiliations:** 1 School of Medicine and Health Sciences, University of Papua New Guinea, Port Moresby, Papua New Guinea; 2 Australian Venom Research Unit, Department of Biochemistry and Pharmacology, Faculty of Medicine, Dentistry and Health Sciences, University of Melbourne, Melbourne, VIC, Australia; 3 Pacific Adventist University, Port Moresby, Papua New Guinea; 4 National Department of Health, Port Moresby, Papua New Guinea

**Keywords:** epidemiology, Papua New Guinea, public health, snakebite, snakebite envenoming, antivenom

## Abstract

**Background:**

Snakebite envenoming is a major public health challenge across Papua New Guinea (PNG). Since 2018, the PNG Snakebite Partnership has distributed life-saving Australian antivenoms to health centres across PNG, trained healthcare workers in appropriate snakebite patient management, and collected snakebite patient data to map the impact of snakebite at participating health centres. Here, we report on the snakebite patient data collected through the PNG Snakebite Partnership between 2018 and 2023.

**Methods:**

This retrospective analysis reviewed the clinical records of patients treated for snakebites at 49 health centres across PNG between 5^th^ March 2018 and 31 December 2023 collected on behalf of the PNG National Department of Health (NDoH) through the PNG Snakebite Partnership. Data included 1. Patient demographics (*e.g.*, age, sex), 2. Circumstances of the bite (*e.g.*, location, size, and time), 3. Observed clinical symptoms, 4. Treatments administered (*e.g.*, antivenom), and 5. Patient outcomes, where available.

**Findings:**

A total of 2,573 snakebite patients attended participating health facilities during the 5.75-year study period. Most snakebite patients were male (59.89%) with a median age of 24 (16.00-35.00). Children (≤16 years of age) accounted for a quarter of all snakebite patients (n = 651, 25.30%). September had the highest number of bites recorded (n = 260) though no statistical differences were observed across months or seasons. Of the 2,573 snakebite patients assessed, 1,199 patients (46.60%) were administered antivenom, with just under half of patients receiving taipan antivenom (n = 535, 44.62%), followed by polyvalent antivenom (n = 442, 36.86%), and death adder antivenom (n = 222, 18.51%). Antivenom was shown to be well tolerated with <15% (n = 169) of patients recorded as experiencing adverse reactions, most of which were classified as mild. 32 fatal snakebites were recorded during the study period, equating to a fatality rate of 2.36% (adult rate – 2.42%; child rate – 2.56%).

**Interpretation:**

With the increased availability of free and effective antivenoms through the Partnership, consistent and widespread public health interventions can work to reduce the burden of snakebite in challenging and resource-limited environments. These preliminary analyses estimate that between 901–1,100 snakebite patients survived their bites after receiving treatment through the Partnership. These estimates highlight the success of the program; however, it is also important to acknowledge that they are based on incomplete data. For a more accurate accounting of the impact of this program, further efforts that increase the collection of accurate and complete patient data are required to provide a more finely tuned assessment of both the scale of snakebite and the impact of the Partnership across PNG.

## Introduction

Papua New Guinea (PNG) is reported to have one of the highest regional incidence rates of snakebite in the world (Williams et al., 2007), and mortality rates from snakebite envenoming are estimated to be almost 100 fold higher than those observed in Australia (McGain et al., 2004). In Central Province, incidence rates have been estimated at 215.5 snakebites per 100,000 people, with individual districts reporting incidence rates over 520 snakebites per 100,000 (Lalloo et al., 1995a). While over 100 species of snake inhabit the lands and coastal waters of PNG (Williams, 2005), the Papuan taipan (*Oxyuranus scutellatus*) and the New Guinea death adders (*Acanthophis rugosus* and *Acanthophis laevis*) are the most medically significant (Lalloo et al., 1995b; Currie, 2000). In the southern coastal regions of Central Province, the Papuan taipan is reportedly responsible for >80% of recorded cases of snakebite envenoming (Lalloo et al., 1995a). Death adders were responsible for ∼10% of bites in Central Province (Lalloo et al., 1995a), however, as the most widely distributed venomous snake in PNG (Lalloo et al., 1996), their clinical impact extends to even the most remote regions of the country. The New Guinea small-eyed snake (*Micropechis ikaheca*) is widely distributed across Papua New Guinea and is also capable of delivering fatal bites (Pla et al., 2014; Warrell et al., 1996). While it only accounts for a relatively small proportion of bites on PNG’s mainland, it is reported to account for up to 40% of snakebites on Karkar Island (Williams, 2005; Warrell et al., 1996). Bites have also been recorded by brown snakes (*Pseudonaja textilis*), and Papuan blacksnakes (*Pseudechis papuanus*); however, bites by these snakes are reported at lower rates (Lalloo et al., 1995a; Lalloo et al., 1995b; Lalloo et al., 1996).

Taipans, death adders, and brown snakes are elapids that evolved in Australia and subsequently radiated North to PNG (Wüster et al., 2005). The New Guinea small-eyed snake, on the other hand is a “Melanesian” elapid–a member of a lineage established in New Guinea during the initial eastward expansion of the elapid snakes from Asia towards Australasia (Paiva et al., 2014). The venom of Papuan elapids contains a mix of neurotoxins, haemotoxins, and in some cases myotoxins; however, neurotoxins are typically considered the major contributors in fatal snakebites (Currie, 2000; Lalloo et al., 1996; Kuruppu et al., 2008; Wi et al., 2005). While varied across species, these neurotoxins act to produce a progressive ‘descending’ flaccid paralysis that suppresses normal respiratory function. The effects of post-synaptic neurotoxins, such as those found in death adder venom, are often reversable by antivenom (Isbister, 2006). However, presynaptic neurotoxins, such as those found in taipan venom, bind irreversibly, and result in the destruction of nerve endings (Isbister, 2006; Johnston et al., 2017). While the early administration of antivenom can prevent paralysis and clinical progression to the respiratory system (Isbister, 2006; Johnston et al., 2017), for moderate to severe envenoming, airway protection and timely intubation are required to ensure patient survival (Lalloo et al., 1995a; Lalloo et al., 1995b). The combination of early antivenom administration and appropriate airway management therefore offers an effective means of significantly mitigating the impact of snakebite envenoming in PNG.

The close phylogenetic relationship between medically significant snake species across PNG and Australia means that Australian antivenoms are effective at neutralising the venoms of most of PNGs venomous elapids (Williams and Wüster 2005; Wüster et al., 2005). For example, monovalent CSL Taipan antivenom and CSL Death Adder antivenom effectively neutralise the venoms of their respective target species in PNG (Trevett et al., 1995a), while CSL Polyvalent antivenom is thought to be effective against bites from all medically significant snake species in PNG (Pla et al., 2014). Despite the efficacy of Australian antivenoms, access to these lifesaving medicines for patients in PNG has been limited. Previous studies have reported that local antivenom supplies were only enough to treat 14.1% of recorded snake bite patients and as few as 7.2% of snakebite patients were actually administered antivenom (Williams et al., 2007). A review of Health Department records also indicated that less than 50% of issued antivenom supplies were accounted for (Williams et al., 2007).

In 2018, Australian antivenom manufacturer Seqirus (a CSL Company), the National Department of Health (NDoH; PNG), and the Australian Government, established the PNG Snakebite Partnership to improve the treatment of snakebite in PNG. This partnership involved a donation, from CSL Seqirus, of ∼600 vials of Australian antivenoms per year to increase access to antivenoms, as well as funding from the Australian Government, through the PNG-Australia Transition to Health (PATH) program, to train healthcare workers in appropriate snakebite patient management, and to establish snakebite patient reporting systems and collect epidemiological data on behalf of the NDoH regarding the impact of snakebite across PNG. Our team managed the in-country work of the Partnership between 2018 and 2024, through a collaboration between the School of Medicine and Health Sciences at the University of PNG and the Australian Venom Research Unit at the University of Melbourne. During this time, >2,725 vials of antivenom and >5,000 patient forms were distributed to >73 health facilities across Papua New Guinea.

Here, we report, for the first time, a retrospective analysis of epidemiological data collected through the PNG Snakebite Partnership. This investigation brings together snakebite patient data from 49 health facilities and 11 Provinces across PNG between 2018 and 2023 in one of the largest investigations into the impact of snakebite undertaken in PNG to date. This work aims to build on previous investigations of snakebite in PNG (McGain et al., 2004; Lalloo et al., 1995a; Trevett et al., 1995a; Currie et al., 1991) by providing a contemporary summary of the scale of snakebite as a public health challenge in PNG and highlighting the role that freely available and effective antivenoms play in addressing this neglected tropical disease.

## Methods

This study involved the retrospective analysis of data extracted from 2,665 snakebite patient forms collected through the PNG Snakebite Partnership on behalf of the National Department of Health (NDoH), PNG.

### Data source

The NDoH PNG Snakebite Database collates patient data from a network of >73 health facilities across 13 provinces in PNG. Data consisted of patient forms pertaining to individuals seeking clinical management of suspected snakebite and/or snakebite envenoming. Paper forms were collected and digitised by transcribing the data into a Microsoft Access database as part of the PNG Snakebite Partnership to form the PNG Snakebite Database. Suspected snakebites include any injury that a patient reports as being a possible snakebite and may include non-snakebite injuries sustained in the presence/vicinity of a snake (*e.g.*, standing on a stick, known as a ‘stick bite’), bites from non-venomous snakes (*i.e.*, pythons, boas, file snakes, *etc.*), bites from venomous snakes not resulting in clinical envenoming (i.e., ‘dry bites’), or bites from venomous snakes resulting in clinical envenoming. The data analysed here included all snakebite patient data collected and digitised by the Partnership for patients attending health centres between 5^th^ March 2018 and 31 December 2023. Data was accessed on the 24 July 2024 as an exported Microsoft Excel file. At this time, data had been collected and digitised from 49/73 (67.12%) health centres to date; however, it is noted that this data may not account for all cases presenting to each respective health facility across the entire study period.

### Patient data

Inclusion criteria included all patients reporting to one of the 73 health centres and/or hospitals supplied with antivenoms and/or patient forms by the PNG Snakebite Partnership between 5^th^ March 2018 and 31 December 2023 (Figure 1). Exclusion criteria included patients receiving non-snake antivenoms (e.g., stonefish antivenom) and duplicate patient records.

**FIGURE 1:**
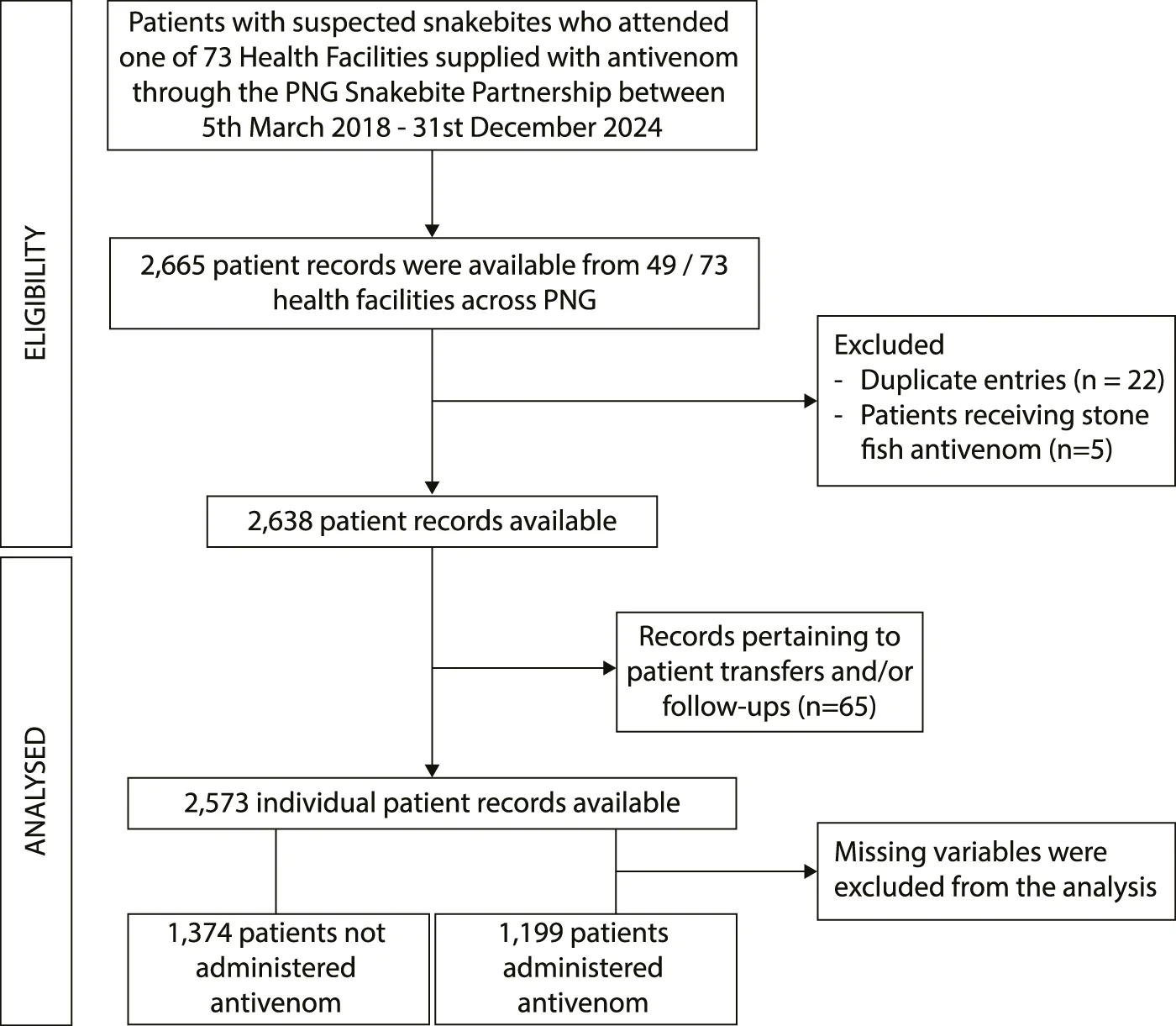
Overview of eligible snakebite patient data included in the analysis.

Each patient record contained data on the following variables: 1. Demographic information, 2. Place and activity of suspected snakebite, 3. Snakebite details including bite site, bite site appearance, and description of snake, if seen, 4. First aid performed, 5. Clinical signs and symptoms, including signs of envenoming, 6. Diagnosis, 7. Treatments administered, including premedications, antivenom, and treatments and details for adverse reactions and complications, and 8. Patient outcomes.

The PNG Snakebite Database contained a total of 2,665 snakebite patient records across the study period. Following a review, duplicate entries (n = 22) and patient transfers/follow-ups (n = 65) were identified alongside 5 patients who received antivenom for stonefish envenoming. Duplicates were identified by matching data bite and treatment data (e.g., date and time of bite, presentation at health centre and treatment, bite location, etc.) alongside health centre visited and basic demographics (e.g., age and sex). Follow-ups were identified by an overlap in these datapoints in addition to new treatment information provided on forms completed at the same health centre. Transfers were identified using the data noted above for duplicates that were completed at nearby health facilities and/or records with direct reference to patient transfers. The remaining data pertained to 2,573 individual snakebite patients. Duplicate records and records pertaining to stonefish envenoming were excluded from the analyses. Data from both initial presentation and follow-ups/transfers were used to identify treatment with antivenom, premedication use, adverse reactions, the treatment of adverse reactions. Antivenom type, time of treatment, batch numbers and other information was used to ensure duplication of antivenom administration was excluded from analyses. Patient outcomes where available were only included from follow-up or transfer forms.

All patient data were analysed, however information pertaining to demographic and geographic data were suppressed in cases where fewer than 10 data points were available, and all data were suppressed in cases where fewer than 5 data points were available, in accordance with our ethics approvals and to ensure the privacy of patients. Missing data values were excluded from the analysis.

#### Patient classification

Patients were classified as children (≤16 years of age) or adults (>16 years of age), as individuals >16 years of age are generally cared for by adult rather than paediatric physicians in PNG (Pameh, 2016). First aid data pertained to the first aid measures applied to the patient prior to them attending the health facility. In instances where multiple measures were used, the first measure noted was included in the analysis.

### Clinical determinants of envenoming

The clinically significant sequelae of envenoming by elapids in PNG are predominantly caused by the actions of haemotoxins and neurotoxins. Evidentiary markers of envenoming from these toxins are provided below, as noted by (McGain et al., 2004; Jensen, 2005).

***Evidence of coagulopathy***: Evidence of coagulopathy was from either a positive 20-min whole blood clotting test (20WBCT) or from spontaneous bleeding from the bite site, gums, or other cuts and abrasions, haematemesis, and haematuria.

The 20WBCT is a test in which ∼2 mL of freshly collected blood is collected in a clean, dry glass vial and left to stand at room temperature for 20 min. A positive 20WBCT, indicative of coagulopathy, is recorded if the blood fails to clot after the elapse of the measurement period. Note: if there is any clotted blood in the glass vial, the blood is considered clotted and a negative 20WBCT is recorded, as noted by (White et al., 2019).

***Evidence of neurotoxicity***: Clinical presentation of venom-induced neurotoxicity is initially observed in smaller muscles of the eyes and face, resulting in symptoms such as ptosis, dysphagia and ophthalmoplegia. Subsequently, the paralysis ‘descends’ to impact the larger muscles of the shoulder, throat, and neck, resulting in dysphagia, and impairing the ability to shrug or turn one’s head. Eventually, in the absence of timely intervention, the musculature of the respiratory system is affected, resulting in dyspnoea and the incorporation of accessory muscles of respiration. Ultimately, neurotoxic envenoming by Papuan elapids may lead to a profound flaccid paralysis, the primary cause of fatal snakebite in the region.

### Diagnosis of snakebite envenoming

Clinicians and healthcare workers used knowledge of venomous snakes in the region, snake descriptions, and symptoms of clinical envenoming to guide their diagnosis of envenoming from specific snake species and subsequent antivenom selection. As direct evidence of the specific snake species involved was often not captured by the patient forms, the administration of monovalent antivenoms was used as a surrogate marker for potential envenoming by specific snake species in the current study. Reference to death adder and taipan antivenom or snakebite in the results should thus be considered as treatment-defined groups rather than confirmed species-specific envenoming.

### Antivenoms administered

Australian antivenom donated by CSL Seqirus through the PNG Snakebite Partnership included: CSL Death Adder antivenom, CSL polyvalent antivenom, CSL Sea Snake antivenom, and CSL Taipan antivenom. All reference to antivenoms administered throughout these analyses refer to these antivenoms.

### Grading adverse reactions to antivenom

The severity of adverse reactions (generalised hypersensitivity reactions) following antivenom administration was based on the scale outlined in (Brown, 2004; Isbister et al., 2008). Here, the scale was adjusted to account for clinical difficulties experienced across PNG relating to airway management. This resulted in an increased severity grading, from moderate to severe, for hypersensitivity reactions involving the airways or respiratory system, including dyspnoea, chest tightness, airway narrowing, and breathing difficulties. Briefly, mild reactions predominantly affected the skin and subcutaneous tissues (i.e., pruritus, urticaria, swelling), in addition to restlessness and irritability; moderate reactions featured more systemic involvement (i.e., nausea/vomiting, signs of fever, diaphoresis, coughing, sneezing); severe reactions included respiratory or neurological signs (i.e., dyspnoea, airway narrowing, chest tightness, breathing difficulties, speech difficulties, seizures).

#### Population data

The snakebite patient data analysed in the current study comes from an estimated catchment population of 2,146,720 individuals. Population data was accessed via the feature layer from ESRI accessed via ArcGIS PRO (3.3.3) titled ‘Papua New Guinea District Boundaries’ with credits to ESRI, Michael Bauer Research GmbH 2022, National Statistical Office of Papua New Guinea. District level snakebite case rates were determined using recorded patient cases presenting to a district health centre/the district population. District level recorded antivenom rates were recorded using recorded antivenom administered at a district health centre/the district population. These rates were then normalised to rates per 100,000 population. District level data was combined to create provincial level insights.

### Statistical analysis

Data were prepared, analysed, and graphed using Microsoft Excel, GraphPad Prism (10.2.3), and ArcGIS PRO (3.3.3). Two-tailed Chi square goodness of fit tests were used to assess differences between rates of snakebite and antivenom between sexes with expected values of 50% utilised for the calculations. While a Chi-square test of independence (two-tailed) was used to compare whether the proportion of men and women receiving antivenom was significantly different than those who did not. Normality was assessed using the Shapiro-Wilk test, t-tests were used to assess differences across seasons, and multiple variate analysis was conducted using ANOVA with Tukey’s multiple comparison tests or Kruskal–Wallis Test. ANOVA was used to assess differences in the numbers of snakebites across the year and differences in time-to-treatment for patients experiencing adverse reactions to antivenoms. Kruskal–Wallis testing was performed to assess differences in the number of antivenom vials administered across the year.

### Supplementary results

Data pertaining to snakebites by time of day, description of snake, and activity undertaken at time of bite are available in the Supplementary Material.

### Ethics statement

This retrospective study utilised an existing snakebite patient database pertaining to suspected snakebite patients across PNG. Data in the database was collected through the PNG Snakebite Partnership on behalf of the NDoH and was retrospectively accessed on the 24 July 2024. Given the retrospective nature of this work, a waiver of consent and ethics were approved by the Medical Research Advisory Committee (24.17/2024) and the University of Melbourne Human Research Ethics Committee (2024-29623-54449-2).

## Results

### Patient demographics

***Suspected snakebite patients***: A total of 2,573 individuals attended health facilities across PNG for treatment of a suspected snakebite during the study period (Table 1; Figure 2). Males (n = 1,544/2,573, 59.89%; median age 23 years, IQR 16.00-34.00) presented to health centres for suspected snakebite at significantly greater rates than females (n = 998/2,573, 38.79%; median age 24 years, IQR 16.00-36.00; χ^2^ = 117.276, df = 1, p < 0.0001). The overall median age of patients was 24 years of age (IQR 16.00-35.00; data available n = 2,483). Children (≤16 years of age; median age 12 years, IQR 8.00-15.00) accounted for a quarter of all snakebite patients (n = 651/2,573, 25.30%), with boys (n = 391/651, 60.06%) accounting for a greater proportion of patients ≤16 years than girls (n = 260/651, 39.94%; χ^2^ = 26.361, df = 1, p < 0.0001).

**TABLE 1:** Demographics of snakebite patients and patients administered antivenom at health centres across Papua New Guinea between 2018 and 2023.

Patients	Male		Female		Unrecorded		Total
	n	%	n	%	n	%	n
Suspected snakebite patients							
Total patients^***^	1,544	60.01	998	38.79	31	1.20	2,573
Adult patients (>16years)^***^	1,109	60.53	723	39.47	-	-	1,832
Child patients (≤16years)^***^	391	60.06	260	39.94	-	-	651
Patients administered antivenom							
Total patients^***^	749	62.47	439	36.61	11	0.92	1,199
Adult patients (>16years)^***^	495	61.19	313	38.69	-	-	809
Child patients (≤16years)^***^	231	65.63	121	34.38	-	-	352

* **, and *** denote p-values of < .05, <.01, and <.001 respectively for Chi-Square Goodness of fit comparisons across gender | % given of row totals.

**FIGURE 2:**
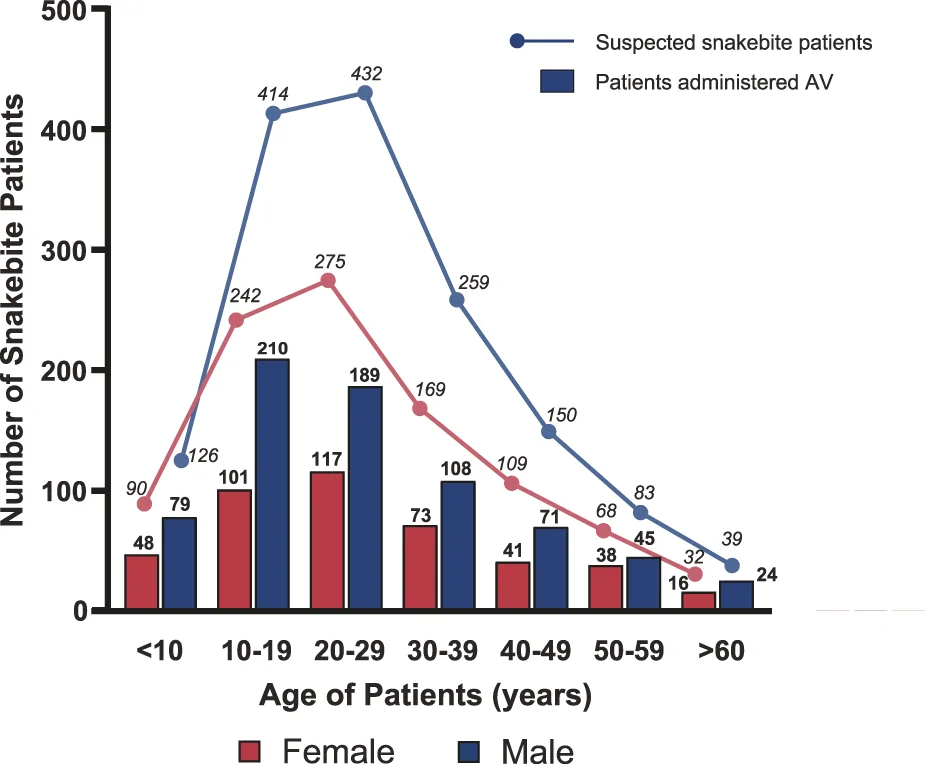
Snakebite patients attending health facilities and administered antivenoms at health facilities across PNG (2018-2023). Data aggregated by age and sex. Line graph shows patients attending health facilities with suspected snakebites. Bar graph shows patients administered antivenom. Data pertaining to patients with no age data recorded not shown.

**Patients administered antivenom**: 1,199/2,573 snakebite patients (46.60%) were administered 1,226 vials of antivenom during the study period (Table 1; Figure 2). Significantly more male patients (n = 749/1,199, 62.47%) were administered antivenom than female patients (n = 439, 36.61%; χ^2^ = 80.892, df = 1, p < 0.0001) overall. Significant differences were also observed in the proportion of snakebite patients receiving antivenom based on sex (male patients – 48.58%, female patients – 43.99%, χ^2^ = 4.980, df = 1, p = 0.0256), with men showing a relative risk of receiving antivenom of 1.1 (1.01,1.20 95% CI). The median age of patients receiving antivenom was 23.5 years of age (15.00-35.00).

Children (≤16 years of age) represented almost a third of snakebite patients receiving antivenom 352/1,199 (29.36%, median age 11 years, 8.00-14.00), with boys (n = 231/352, 65.63%) accounting for a greater proportion of patients ≤16 years receiving antivenom than girls (n = 121/352, 34.38%; χ^2^ = 34.375, df = 1, p < 0.0001). Significant differences were also observed in the proportion of snakebite patients ≤16 years receiving antivenom based on sex (boys – 59.08%, girls – 46.54%, χ^2^ = 9.889, df = 1, p = 0.0017), with boys showing a relative risk of receiving antivenom of 1.27 (1.09-1.48 95% CI) compared to girls.

#### Type of antivenom administered

Of the patients receiving antivenom, 535/1,199 received taipan antivenom (44.62%), 442/1,199 received polyvalent antivenom (36.86%), and 222/1,199 received death adder antivenom (18.51%; Figure 3). No patients were recorded as being administered sea snake antivenom during the study period. Antivenom type noted above is for first vial administered for patients receiving multiple vials.

**FIGURE 3:**
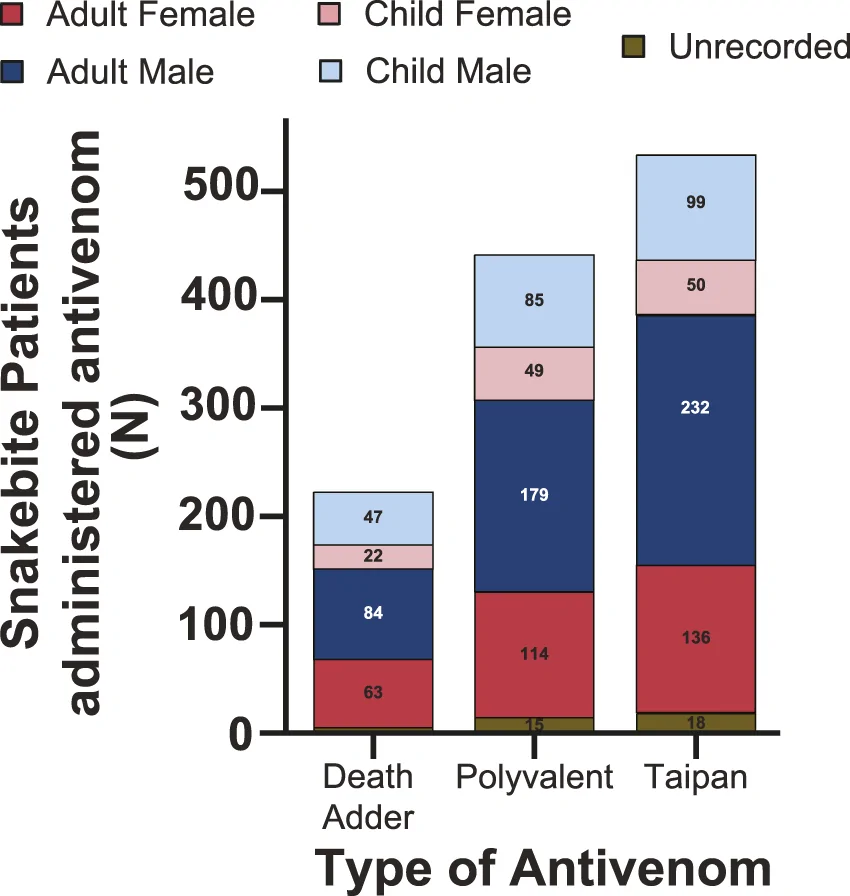
Type of antivenom administered to snakebite patients across PNG (2018-2023). Data aggregated by age and sex.

Administration of multiple vials of antivenom: 26/1,199 patients (2.17%) were administered a second vial of antivenom; 11 female patients (2.51% of the 439 female patients receiving antivenom) and 15 male patients (2.00% of the 749 male patients receiving antivenom). 14 patients (1.16% of the 1,199 patients receiving antivenom) received a vial of taipan antivenom following an initial vial of either polyvalent (n = 7/1,199, 0.58%) or taipan antivenom (n = 7/1,199, 0.58%). 5/1,199 patients received two vials of polyvalent antivenom (0.42%). The remaining 7/1,199 patients received death adder antivenom in conjunction with either taipan or polyvalent antivenom (0.58%).

### Snakebites by district and province

Data on the province and district where healthcare was sought following a suspected snakebite were available for 2,159/2,573 patients (83.91%). Snakebite patients attended health facilities across 10 provinces between 2018 and 2023 (Table 2). Almost 3 quarters of snakebite patients attended health facilities within Central Province (n = 1,574/2,159, 72.90%). The remaining quarter sought treatment across East Sepik, Eastern Highlands, Gulf, Madang, Milne Bay, Morobe, Oro, and Western Provinces (n = 585/2,159, 27.12%). Data for West Sepik was not shown as counts for snakebites and antivenom administrations were <10.

**TABLE 2:** Snakebite patients and antivenom administered by Province and district (2018-2023). Number of patients (count), percentage of patients and recorded case rates of snakebite and antivenom administration reported. District level population data was accessed via the feature layer from ESRI accessed via ArcGIS PRO (3.3.3) titled ‘Papua New Guinea District Boundaries’ with credits to ESRI, Michael Bauer Research GmbH 2022, National Statistical Office of Papua New Guinea.

Province	District	Population	Snakebite patients			Patients administered antivenom		
			Count	%	Recorded case rate	Count	%	Recorded case rate
Central	Abau	68,021	155	7.18	227.87	75	7.43	110.26
Central	Kairuku-hiri	148,832	453	20.98	304.37	119	11.79	79.96
Central	National capital district	445,720	646	29.92	144.93	344	34.09	77.18
Central	Rigo	69,172	320	14.82	462.61	172	17.05	248.66
East sepik	Wewak	107,427	103	4.77	95.88	77	7.63	71.68
Eastern highlands	Goroka/Henganofi	202,236	34	1.57	16.81	31	3.07	15.33
Gulf	Kerema/Kikori	193,166	88	4.08	45.56	64	6.34	32.10
Madang	Madang	135,417	49	2.27	36.18	10	0.99	7.38
Milne bay	Alotau	121,844	63	2.92	51.71	34	3.37	27.90
Morobe	Lae	181,216	37	1.71	20.42	-	-	-
Oro	Ijivitari	122,117	18	0.83	14.74	-	-	-
Oro	Sohe	105,941	58	2.69	54.75	-	-	-
Western	Middle fly	97,130	72	3.33	74.13	29	2.87	29.86
Western	North fly	76,364	48	2.22	62.86	34	3.37	44.52
Western	South fly *	72,117	15	0.69	20.80	-	-	-
**Total**	​	**2,146,720**	**2,159**	**100.00**	**100.71**	**1,009**	**100.00**	**47.00**

* South Fly data includes data from YWAM, Medical Ships and SDP, Aerial Health Patrol. | Total counts include values < 10 that have been removed to maintain privacy. | Recorded case rate - figures given are recorded patient cases presenting to a district health centre/100,000 people population of the district. Totals are provided in bold.

Case recorded snakebite rates across districts: District-level case recorded rates ranged from 14.74 snakebites per 100,000 people in Ijivitari, Oro Province to 462.61 snakebites per 100,000 people in Rigo, Central Province. The overall case recorded rate was 100.71 snakebites per 100,000 people, with an annual case rate of 16.79 snakebites per 100,000 people per year. Demographic data for snakebites by Province is provided in Supplementary Table S1.

Antivenom administered by district and Province: Snakebite patients were administered antivenom at health facilities across 10 provinces between 2018 and 2023: Central, East Sepik, Eastern Highlands, Gulf, Madang, Milne Bay, Morobe, Oro, West Sepik and Western provinces (Tables 2, 3; Figure 4). Data on the district/province where antivenoms were administered following a snakebite was available for 1,009/1,199 patients (84.15%). 70% of patients were administered antivenom at health facilities in Central Province (n = 710/1,009, 70.37%). The remaining patients received antivenom across East Sepik, Eastern Highlands, Gulf, Madang, Milne Bay, Morobe, Oro, West Sepik and Western provinces (n = 299/1,009, 29.63%).

**TABLE 3:** Snakebite patients administered antivenom by province between 2018 and 2023.

Province	Death adder		Polyvalent		Taipan		Total	
	Count	%	Count	%	Count	%	Count	%
Central	81	44.51	201	53.74	428	94.48	710	70.37
East sepik	31	17.03	46	12.30	-	-	77	7.63
Eastern highlands	25	13.74	-	-	-	-	31	3.07
Gulf	-	-	54	14.44	-	-	64	6.34
Madang/Morobe	-	-	15	4.01	-	-	20	1.98
Milne bay/Oro	-	-	22	5.88	12	2.65	34	3.37
West sepik/Western	33	18.13	28	7.49	12	2.65	73	7.23
**Patients with provincial data**	***182***	***81.98***	***374***	***84.62***	***453***	***84.67***	**1,009**	**84.15**
Unrecorded	*40*	*18.02*	*68*	*15.38*	*82*	*15.33*	190	15.85
**Total**	**222**	**-**	**442**	**-**	**535**	**-**	**1,199**	-

% of antivenom usage by province based on number of patients with provincial data | % of ‘Patients with provincial data’ and ‘Unrecorded’ based on Total antivenom usage. Available patient data figures and totals are provided in bold.

**FIGURE 4:**
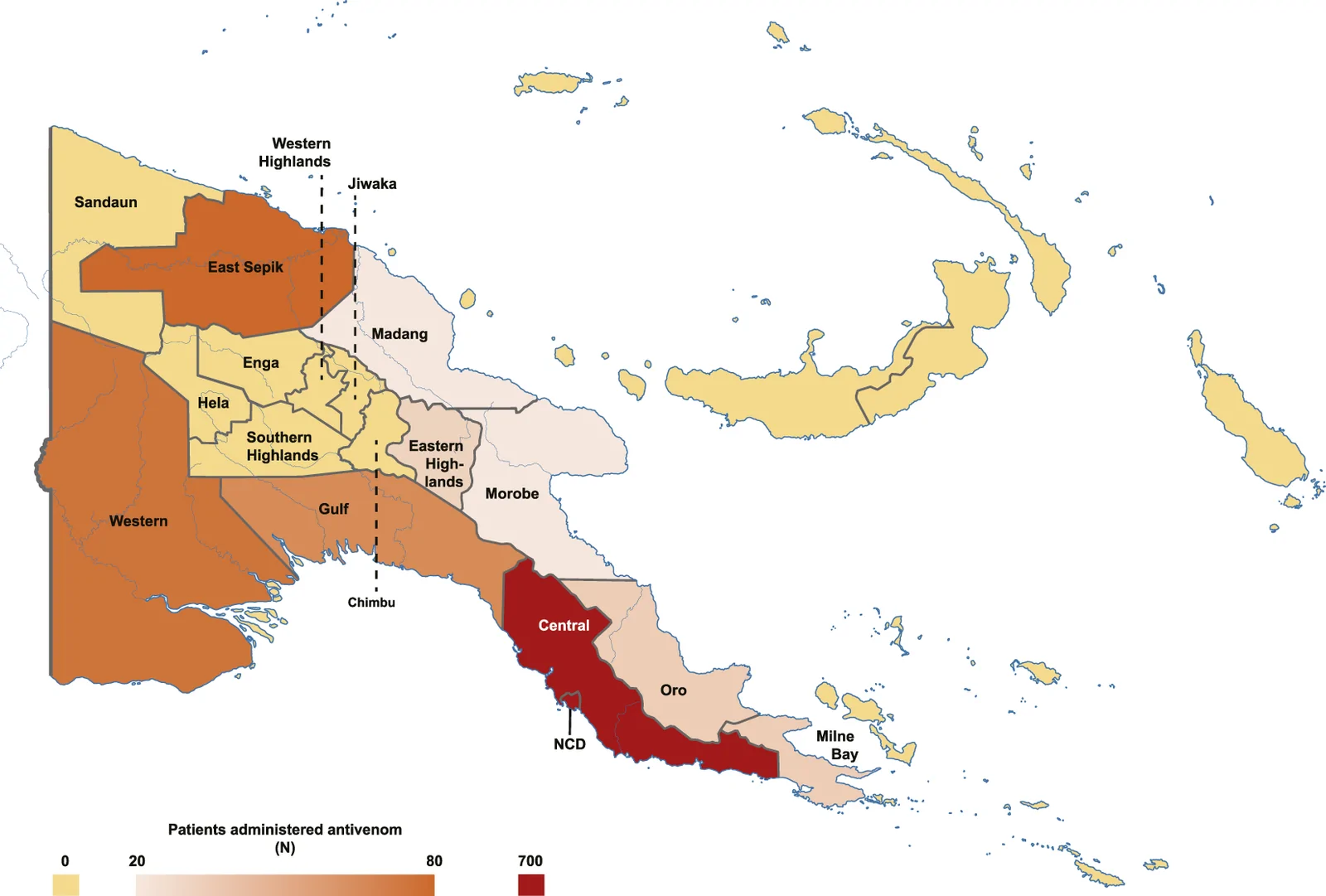
Provincial level map of where snakebite patients were administered antivenom across PNG (2018-2023). Provincial data for Madang and Morobe, and Milne Bay and Oro are combined. This map is an amended map of PNG highlighting the rate of antivenom usage during 2018–2023 and was created using the Papua New Guinea location map by NordNordWest under the Creative Commons Attribution-Share Alike 3.0 Unported license (https://creativecommons.org/licenses/by-sa/3.0/deed.en).

Of the data with known provincial usage, approximately 95% of taipan antivenom was administered in Central Province (n = 428/453, 94.48%; Table 3), with the remaining vials administered in facilities across Milne Bay, Oro, and Western provinces. Central province also accounted for >50% of the polyvalent antivenom administered (n = 201/374, 53.74%) and almost 45% of the death adder antivenom administered (n = 81/182, 44.51%).

Approximately 1 in 6 patients receiving antivenom did not have data relating to where they received antivenom recorded (n = 190/1,199, 15.85%). This included 40/222 patients administered death adder antivenom (18.02%), 68/442 patients administered polyvalent antivenom (15.38%) and 82/535 patients administered taipan antivenom (15.85%).

Recorded antivenom case rates: Recorded rates of antivenom administration by district ranged from 7.38 patients administered antivenom per 100,000 people in Madang (Madang Province) to 248.66 patients administered antivenom per 100,000 people in Rigo, Central Province (Table 2). The overall antivenom case rate for the study period was 47.00 patients administered antivenom per 100,000 people, with an annual rate of 7.83 patients administered antivenom per 100,000 people per year.

### Bites by time of year

While suitable variations in the number of snakebite patients presenting to health facilities and receiving antivenom were observed across the months (Figure 5), no significant differences were observed between the number of bites recorded per month (ANOVA, F = 0.5567, p = 0.4433) nor for the number of vials of antivenom administered per month (Kruskal–Wallis test, Kruskal–Wallis statistic = 10.24, p = 0.5089). Significant differences were also not observed across seasons for the number of bites (t = 1.8285, df = 10, p = 0.0974) nor for the number of antivenoms administered (t = 1.4096, df = 10, p = 0.1890), using previously defined ‘rainy’ (November to April) and ‘dry” (May to October) seasons (Currie et al., 1991). On average, 1.21 people sought treatment for snakebite every day during the study period and a vial of antivenom was administered every 2 days. Data on the month that snakebite patients attended facilities and were administered antivenom was available for 2,548/2,573 (99.02%) patients and 1,196/1,199 (99.75%) patients, respectively.

**FIGURE 5:**
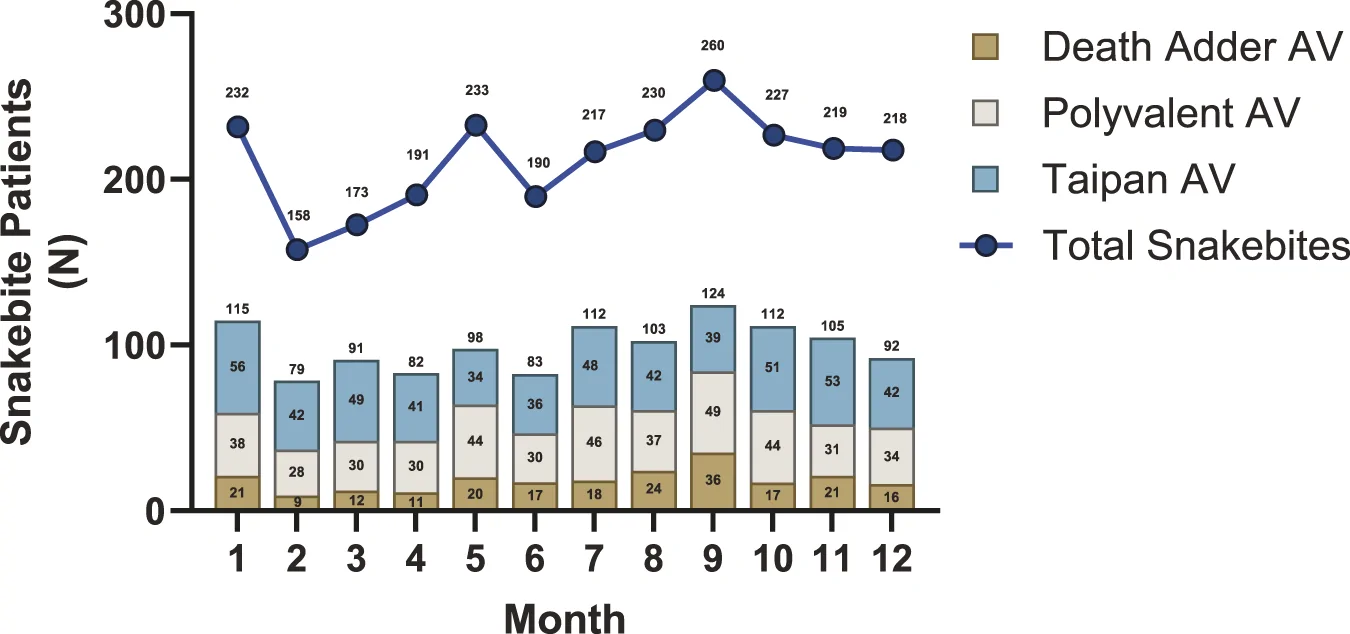
Snakebite patients administered antivenom at health facilities across PNG, 2018-2023. Data disaggregated by month and Antivenom (AV) type. Line graph shows patients attending health facilities with suspected snakebites. Bar graph shows patients administered antivenom.

### Bite site location

Bite site location data was available for 2,187/2,573 (85.00%) snakebite patients. Bites to the feet (n = 1,039/2,187, 47.51%) and lower limbs (n = 1,012/2,187, 46.27%) accounted for >90% of bite (Table 4). Hands, wrists, and/or arms were bitten in just >5% of bites (n = 125/2,187, 5.72%) while bites to other regions, including the groin, torso, and/or head were recorded in only 0.50% of patients (n = 11/2,187).

**TABLE 4:** Bite site location data for snakebite patients across PNG, 2018-2023.

Location of snakebite	Death adder		Polyvalent		Taipan		Total antivenom		TOTAL	
	Count	%	Count	%	Count	%	Count	%	Count	%
Feet	100	47.84	176	47.06	233	50.11	509	48.34	1,039.00	47.51
Ankles/legs	97	46.41	177	47.33	210	45.16	484	45.96	1,012.00	46.27
Hands/wrists/arm	12	5.74	21	5.61	22	4.73	55	5.22	125.00	5.72
Other (groin, torso, head)	-	-	-	-	-	-	5	0.47	11.00	0.50
Patients with bite site data	209	94.14	374	84.62	465	86.92	1,053	87.82	2,187	85.00
Total patients	222	-	442	-	535	​	1,199	​	2,573	​

Total counts include data from unrecorded patients and values < 5 that have been removed to maintain privacy | Total antivenom = total data for patients administered antivenom | TOTAL, All snakebite patient data | % of bite site data based on number of patients with bite site data | % of ‘Patients with bite site data’ based on Total patients.

### Time between snakebite and attendance at a health facility

Data on time between the snakebite occurring and the patient attending a medical facility were available for 1,487/2,573 (57.79%) snakebite patients and 709/1,199 (59.13%) patients administered antivenom. Snakebite patients took a median time-to-treatment of 3 h (IQR 1.50-6.00) to attend a health facility following a bite (Table 5). >25% of patients subsequently administered antivenom attended a health facility within 3 h of their bite (n = 196/709, 27.64%). This figure increased to >60% by 6 h (n = 442/709, 62.34%) and >80% by 9 h post bite (n = 570, 80.39%). By 12 h post bite, >85% of patients subsequently administered antivenom had attended a health facility (n = 618/709, 87.15%).

**TABLE 5:** Time between bite and attendance at health facility.

Time between snakebite and health centre attendance	Death adder		Polyvalent		Taipan		Total antivenom		TOTAL	
	Count	%	Count	%	Count	%	Count	%	Count	%
<1h	11	10.19	18	7.53	19	5.25	48	6.77	190	12.78
1-<3 h s	15	13.89	48	20.08	85	23.48	148	20.87	464	31.20
3-<6 h s	23	21.30	73	30.54	150	41.44	246	34.70	428	28.78
6-<9 h s	18	16.67	46	19.24	64	17.68	128	18.05	167	11.23
9-12 h s	11	10.19	12	5.02	25	6.91	48	6.77	60	4.03
12-<24 h s	-	-	-	-	-	-	11	1.55	20	1.34
24 h s - <48 h s	20	18.52	36	15.06	12	3.31	68	9.59	126	8.47
48 - <72 h s	7	6.48	-	-	-	-	12	1.69	19	1.28
Data available	108	48.65	239	54.07	362	67.66	709	59.13	1,487	57.79
Total patients	222	​	442	​	535	​	1,199	​	2,573	​

Total counts include data from unrecorded patients and values < 5 that have been removed to maintain privacy. | Total antivenom = total data for patients administered antivenom | TOTAL, All snakebite patient data | % of time data based on number of patients with data available | % of ‘Data available’ based on Total patients.

Approximately 70% of patients administered taipan antivenom attended health facilities within 6 h of the snakebite (n = 254/362, 70.17%), compared to ∼60% of patients administered polyvalent antivenom (n = 139/239, 58.15%), and ∼45% of patients administered death adder antivenom (n = 49/108, 45.38%). By 12 h post bite, ∼95% of patients receiving taipan antivenom (n = 343/362, 94.75%), >80% of patients receiving polyvalent antivenom (n = 197/239, 82.43%), and >70% of patients receiving death adder antivenom (n = 78/108, 72.22%) had attended health facilities. Almost 1 in 5 patients administered death adder antivenom (n = 20/108, 18.52%) and 1 in 6 patients administered polyvalent antivenom (n = 36/239, 15.06%) had not attended a health centre until 24–48 h post bite.

## First aid applied to patients prior to arrival at health facilities

First aid data was available for just under 80% of snakebite patients in the database (n = 2,031/2,573, 78.94%). The application of a pressure immobilisation bandage (PIB) is the recommended first aid for venomous elapids in PNG. 17.97% of patients (n = 365/2,031) for whom first aid data was available were recorded as having PIB administered following a snakebite (Figure 6). A further 30.08% (n = 611/2,031) of patients were managed with a pressure bandage without immobilisation. The non-invasive traditional practise of placing a black stone, or snake stone, on the bite site was observed in 67/2,031 patients (3.30%), while the use of other first aid measures (including bush herbs, drinking urine, sucking the bite site, or immobilising the patient without bandaging the bitten limb) were recorded for 31/2,031 patients (1.53%).

**FIGURE 6:**
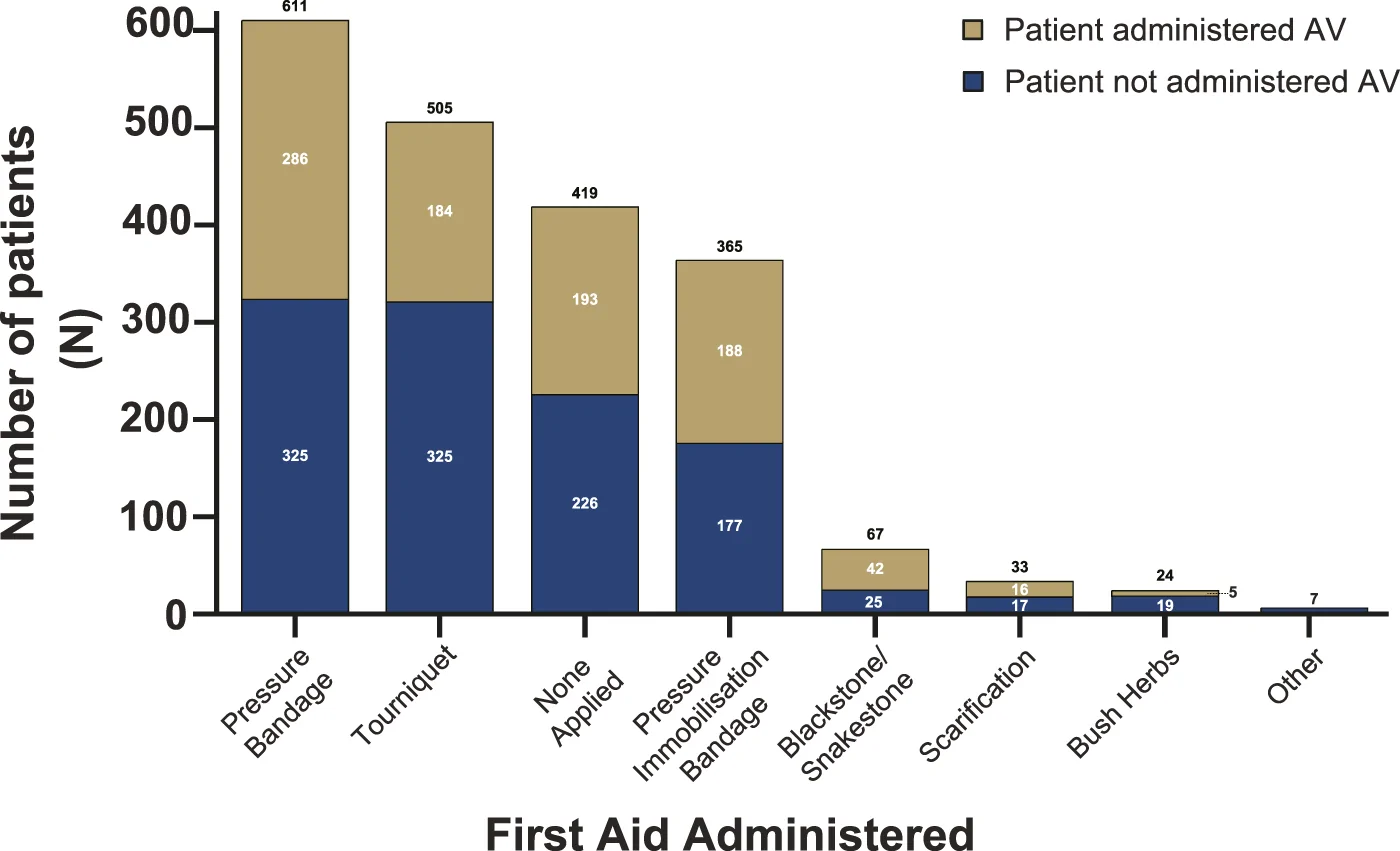
First aid administered to snakebite patients prior to arrival at health facilities for snakebites occurring across PNG, 2018-2023.

Concerningly, more than a quarter of patients had first aid applied that is considered both ineffective for snakebite and harmful to the patient (Bal et al., 2005), including the application of a tourniquet or improvised ligature to the bitten limb (n = 505/2,031, 24.86%) or signs of scarification at the bite site (n = 33/2,031, 1.62%).

## Presenting symptoms of envenomed patients

Approximately 50% of snakebite patients subsequently administered antivenom presented with nausea and/or vomiting (n = 601/1,199, 50.13%) and lymph pain (n = 548/1,199, 45.70%; Figure 7). Abdominal tenderness was observed in a third of these patients (n = 420/1,199, 35.03%), while guarding was recorded in 10% of patients (n = 117/1,199, 9.76%). 24/1,199 patients were recorded as having hepatomegaly and/or splenomegaly (2.00%). Patients not administered antivenoms had observably lower rates of all presenting symptoms.

**FIGURE 7:**
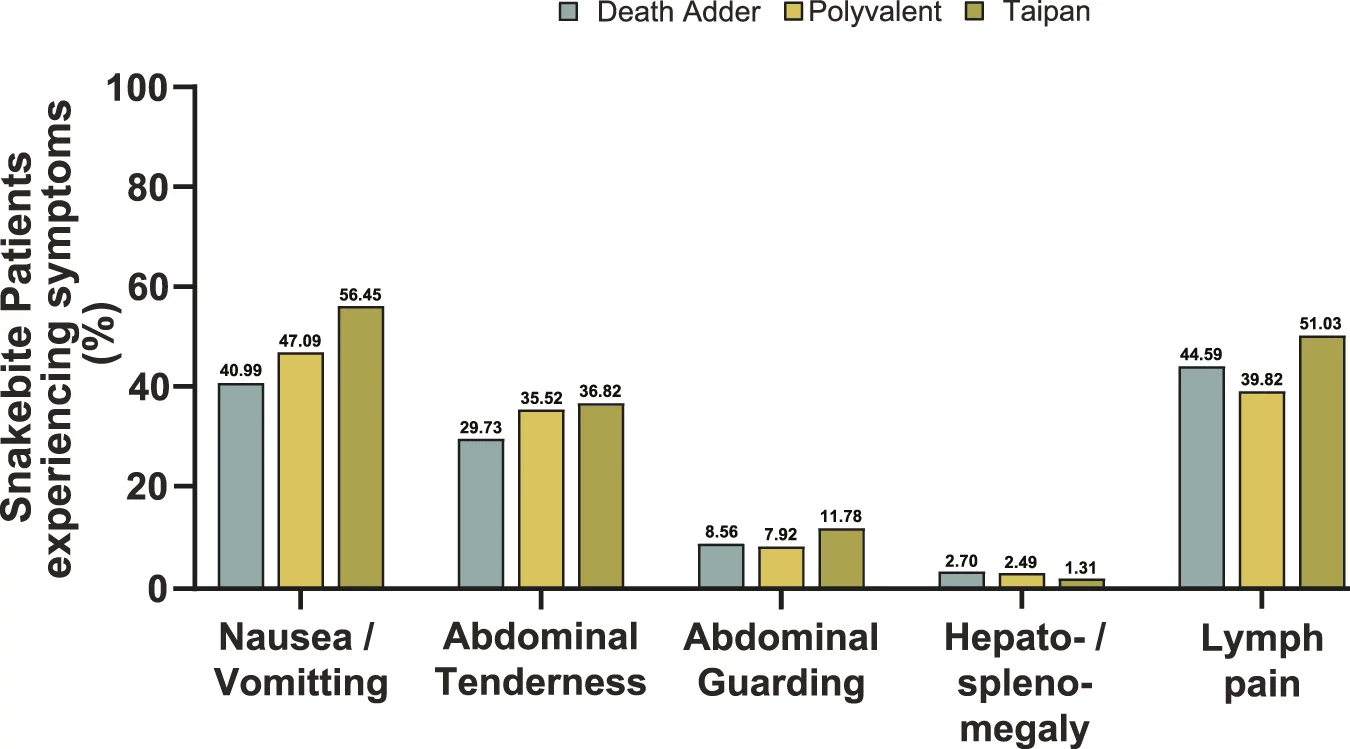
Presenting symptoms of snakebite patients disaggregated by antivenoms administered.

83/1,199 patients (6.92%) were unconscious on arrival at the health facility, and 8/1,199 patients (0.67%) patients were pregnant at the time of their antivenom treatment.

### Evidence of envenoming

**Coagulopathy**: A positive 20-min whole blood clotting test (20WBCT) was the predominant coagulopathic sign for patients receiving antivenom across PNG, with ∼60% of patients recording a positive 20WBCT (n = 744/1,199, 62.05%; Figure 8). Almost 80% of patients receiving taipan antivenom had a positive 20WBCT recorded (n = 425/535, 79.44%), compared to only 35.14% of patients receiving death adder antivenom (n = 78/222). Approximately 15% of patients not administered antivenom were also recorded as having a positive 20WBCT (n = 206/1,374, 14.99%). These results may reflect false positives resulting from improper testing procedures, necessitating additional training, or the presence of mild envenoming, either not requiring antivenom, or cases when antivenom was indicated but no antivenom was available.

**FIGURE 8:**
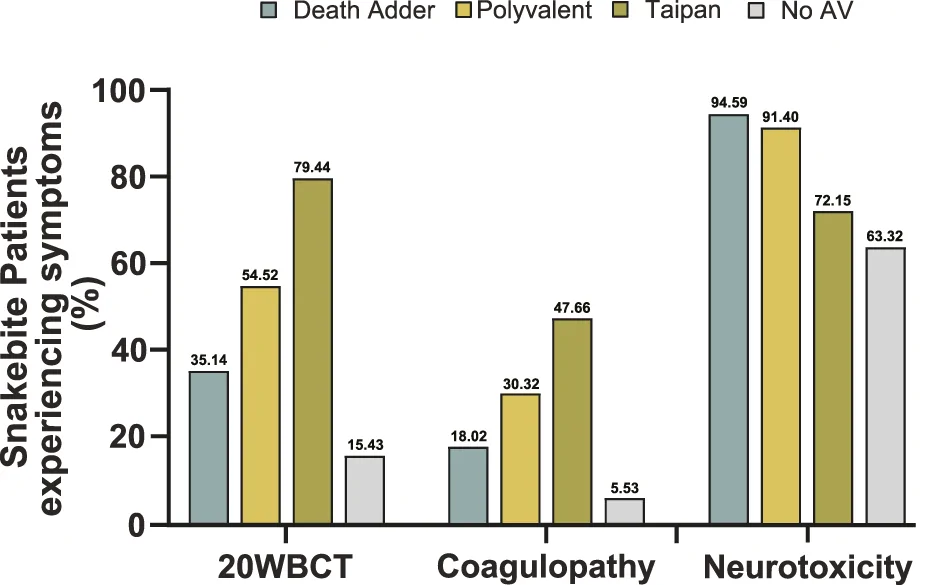
Signs of clinical envenoming disaggregated by antivenoms administered.

Other signs of coagulopathy, including gingival bleeding, spitting blood, bleeding at the bite site, haematemesis, and/or haematuria, were also observed at greater levels in patients administered taipan antivenom. It is noted, that patients recorded as having haematuria may also present with myoglobinuria and underlying myolysis (White, 2018) which has been noted for bites from both small-eyed snake and taipan envenoming (Sitprija, 2006; Warrell et al., 1996). Limited levels of bleeding were observed in patients not administered antivenom.

***Neurotoxicity***: Neurotoxic effects of envenoming were seen in over 80% of all patients receiving antivenom across the study period (n = 1,000/1,199, 83.40%; Figure 8). >90% of patients receiving either death adder (n = 210/222, 94.59%) or polyvalent (n = 404/442, 91.40%) antivenom were recorded as showing signs of neurotoxic envenoming, compared to ∼70% of patients receiving taipan antivenom (n = 386/535, 72.15%). Signs of neurotoxicity included ptosis, ophthalmoplegia, diplopia, and/or the patient’s ability to stick out their tongue, swallow, open their mouths (>2 cm), turn their head, flex their neck, lift their head of the bed, and/or shrug their shoulders. As noted above, the prevalence of symptoms associated with neurotoxicity in patients not administered antivenom may highlight inaccurate reporting, or the presence of mild envenoming. They also act to highlight both the subjective nature of recorded symptoms and the need for ongoing training for healthcare workers particularly in remote regions.

It is noted that the availability of data for patients varied in completeness across these measures and so values shown reflect minimum levels rather than absolute values.

## Diagnosis of snakebite patients

Data relating to clinical diagnosis for patients administered antivenom was available for 1,149/1,199 (95.83%) patients administered antivenom during the study period (Table 6). Snakebite with clinical indications of envenoming were reported as the diagnosis for 837/1,149 patients subsequently administered antivenom (72.85%), with 88/1,149 (7.66%) of these patients recording a diagnosis pertaining to envenoming from a specific snake species; either death adder (n = 60/1,149, 5.22%) or taipan (n = 28/1,149, 2.44%). Snakebite, with no indication of envenoming status, was recorded as the diagnosis in a quarter of cases (n = 290/1,149, 25.24%), while snakebite with no envenoming was recorded for 22/1,149 patients (1.91%) who were subsequently administered antivenom. Just over 10% of patients not administered antivenom (n = 152/1,277, 11.90%) were also diagnosed with snakebite envenoming.

**TABLE 6:** Clinical diagnosis for snakebite patients receiving antivenom across PNG, 2018-2023.

Clinical diagnosis of snakebite patients	Death adder		Polyvalent		Taipan		Total AV		No AV administered	
	*Count*	*%*	*Count*	*%*	*Count*	*%*	*Count*	*%*	*Count*	*%*
Snakebite envenoming	171	82.61	268	62.62	398	77.43	837	72.85	152	11.90
- Snakebite envenoming	80	38.65	173	40.42	201	39.11	454	39.51	80	6.26
- Snakebite with coagulopathy	-	-	19	4.44	88	17.12	107	9.31	9	0.70
- Snakebite with neurotoxicity	44	21.26	30	7.01	27	5.25	101	8.79	7	0.55
*- Snakebite with neurotoxicity and coagulopathy*	-	-	33	7.71	54	10.51	87	7.57	9	0.70
*- Death Adder*	47	22.71	13	3.04	-	-	60	5.22	40	3.13
- Taipan Bite	-	-	-	-	28	5.45	28	2.44	7	0.55
Snakebite	36	17.39	146	34.11	108	21.01	290	25.24	592	46.36
Snakebite (no envenoming)	-	-	14	3.27	8	1.56	22	1.91	509	39.86
Patients with diagnostic data	207	100.00	428	100.00	514	100.00	1,149	100.00	1,277	100.00

### Premedication administration

Patients received premedications before antivenom administration in 923/1,199 instances (76.98%; Supplementary Table S5). Almost two-thirds of patients administered antivenom were premedicated with adrenaline (n = 773/1,199, 64.47%). Adrenaline was predominantly administered via sub-cutaneous injection (n = 681/773, 88.10%), though a small number of patients received adrenaline via intra-venous (n = 44/773, 5.69%) or intramuscular (n = 29/773, 3.75%) injection. 145/1,199 (12.09%) patients administered antivenom were premedicated with hydrocortisone, while 5/1,199 patients received pre-medication with other compounds such as promethazine (0.42%).

#### Adverse reactions including rates, features, premedications and treatments

169/1,199 patients were recorded as having adverse reactions following antivenom administration, an adverse reaction rate of 14.10% (Table 7). While adverse reactions were observed for all antivenoms, patients receiving polyvalent antivenom had the highest rate of reactions (n = 76/442, 17.19%).

**TABLE 7:** Adverse reaction rates, features, premedications and treatments for patients administered antivenom across PNG, 2018-2023.

​	AV - No reaction		Death adder		Polyvalent		Taipan		AV - adv reaction	
	Count	%	Count	%	Count	%	Count	%	Count	%
Patients administered AV	1,199	-	222	-	442	-	535	-	1,199	-
Patients with adverse reactions	1,030	85.90%	23	10.36	76	17.19	70	13.08	169	14.10
Premedication	798	77.48	22	95.65	65	85.53	60	85.71	147	86.98
- Adrenaline	671	65.15	21	91.30	49	64.47	54	77.14	124	73.37
- Hydrocortisone	123	11.94	-	-	16	21.05	5	7.14	21	12.43
Time to treatment (hours)										
- Median (hours)	4.5	-	6.77	-	5	-	3.79	-	4.5	​
- 1st quartile (hours)	2.5	-	5.4	-	3	-	2.12	-	3	​
- 3rd quartile (hours)	7.52	-	8.91	-	8.5	-	5.88	-	7.38	​
- Data available	612	59.42	16	69.57	41	53.95	50	71.43	107	63.31
Severity of reaction										
- Mild	-	-	8	34.78	43	56.58	39	55.71	90	53.25
- Moderate	-	-	7	30.43	5	6.58	7	10.00	19	11.24
- Severe	-	-	5	21.74	9	11.84	9	12.86	23	13.61
- Data available	-	-	20	86.96	57	75.00	55	78.57	132	78.11
Treatment	​	​	7	30.43	40	52.63	29	41.43	76	44.97
- Adrenaline	-	-	-	-	19	25.00	13	18.57	32	18.93
- Hydrocortisone	-	-	-	-	18	23.68	14	20.00	32	18.93

Total counts include data from other premedications/treatments and values < 5 that have been removed to maintain privacy. Data in brackets refers to patients with no adverse reactions noted. | AV, Antivenom |Adv Reaction - Adverse reactions | ‘AV, No reaction’ refers to patients administered antivenom who had no adverse reactions reported.

147/169 (86.98%) patients who experienced adverse reactions received premedication. Almost 3 quarters of these patients received adrenaline (n = 124/169, 73.37%), while a further 21/169 patients received hydrocortisone (12.43%).

Time to treatment data was available for 107/169 (63.31%) patients experiencing adverse reactions. The median time to treatment for these patients was 4.5 h (IQR 3.00-7.38). No significant differences were observed in the time to receive treatment between patients experiencing adverse reactions and those who did not (ANOVA F = 1.43, p = 0.23).

Data on the severity of the reactions was available for over >75% of patients experiencing adverse reactions (n = 132/169, 78.11%). Mild-to-moderate reactions were experienced by almost two-thirds (mild - n = 90/169, 53.25%; moderate–n 19/169, 11.24%), while severe reactions (which included respiratory or neurological signs) were experienced by 23/169 patients (13.61%). No patient deaths were attributable to adverse reactions during the study period.

Treatments for adverse reactions were recorded for less than half of patients experiencing reactions (n = 76/169, 44.97%). These patients received adrenaline (n = 32/169, 18.93%), either alone or in combination with hydrocortisone or promethazine, or hydrocortisone (n = 32/169, 18.93%), either alone or in combination with promethazine, in approximately equal numbers. A small number of patients were treated with other medications such as promethazine or neostigmine (data not shown).

## Response to AV therapy

Patient forms collected responses to antivenom across the following categories: Excellent, Good, and Poor. Responses were determined subjectively by the health worker completed the forms. These criteria were not based on strict clinical indicators nor were they completed at a specific time post treatment. They have been included here to provide an indication into whether patients responded positively (Good or Excellent) or poorly (Poor) to antivenom administration.

Data on patient responses to antivenom administration was available for 684/1,199 patients (57.05%; Table 8). Of these, ∼90% of patients (n = 604/684, 88.30%) were recorded as having a good (n = 358/684, 52.34%) or excellent (n = 246/684, 35.96%) response to the antivenom therapy. 80/684 patients (11.70%) were recorded as having a poor response.

**TABLE 8:** Patient responses to antivenom across PNG, 2018-2023.

​	Death adder		Polyvalent		Taipan		Total	
	*Count*	*%*	*Count*	*%*	*Count*	*%*	*Count*	*%*
Patients administered AV	222	-	442	-	535	-	1,199	-
Data available - AV response	152	68.47	297	67.19	235	43.93	684	57.05
Excellent response	69	45.39	116	39.06	61	25.96	246	35.96
Good response	78	51.32	147	49.49	133	56.60	358	52.34
Poor response	5	3.29	34	11.45	41	17.45	80	11.70

AV, antivenom.

## Patient outcomes following snakebite

***Fatal snakebites***: A total of 32 fatal snakebites were recorded during the study period (Table 9). The median age of patients suffering a fatal bite was 26.50 years of age (IQR 15.00–40.75 years). 22 of the patients were male (68.75%, median age = 26.50, IQR 15.00–42.25). The remaining 10 were female (31.25%, median age = 23.50, IQR 17.00–35.25). Patients (≤16 years of age accounted for just under a third of fatal snakebites (31.25%, median age = 14.00, IQR 10.75–15.00). Case fatality rates were estimated by dividing total fatal snakebites over total patients receiving antivenom (n = 1,199) and total patients diagnosed with snakebite envenoming but who did not receive antivenom (n = 152). This equated to a case fatality rate of 2.36% across the study period. It is noted that due to the lack of certainty regarding the completeness of the patient outcome data, the above rate is viewed as the lower limit of the fatality rate attributable to snakebite across the study period.

**TABLE 9:** Demographic data pertaining to fatal snakebites across PNG, 2018-2023.

All patients	
Number of patients n (%)	32
Patient age Median (IQR)	26.5 (15.00–40.75)
Data available n (%)	32 (100%)
Case-fatality rate	2.36
Male patients	
Number of patients n (%)	22 (68.75%)
Patient age Median (IQR)	26.5 (15.00–42.25)
Case-fatality rate	2.62
Female patients	
Number of patients n (%)	10 (31.25%)
Patient age Median (IQR)	23.5 (17.00–35.25)
Case-fatality rate	2.05
Adult patients (>16 years)	
Number of patients n (%)	22 (68.75%)
Patient age Median (IQR)	35.5 (26.25–52.25)
Case-fatality rate	2.42
Child patients (≤16 years)	
Number of patients n (%)	10 (31.25%)
Patient age Median (IQR)	14.00 (10.75–15.00)
Case-fatality rate	2.56

Case-fatality rates were calculated using patients receiving antivenom plus patients diagnosed with snakebite envenoming but who did not receive antivenom as the denominator.

The median time-to-treatment for fatal snakebites was 5.73 h (IQR 3.00–9.19; Table 10). Pressure bandage without immobilisation was applied to just under half of these patients (n = 12, 46.15%), while approximately 1 in 3 had either a tourniquet or no first aid applied (n = 5, 19.23% for each measure; 31.25% combined). 50% showed signs of coagulopathy and >90% showed signs of neurotoxicity (n = 30, 93.75%). 23 of the patients received antivenom prior to their deaths (71.88%). This overall case fatality rate was 2.36%, based on a denominator of all patients receiving antivenom plus those who received no antivenom but received a diagnosis of snakebite envenoming, and a recorded snakebite mortality rate of 1.49 deaths per 100,000 people (annual recorded mortality rate of 0.25 deaths per 100,000 people per year). It is acknowledged that these figures underestimate the true rate of snakebite deaths across PNG due to incomplete data on case outcomes.

**TABLE 10:** Time to treatment, first aid and clinical signs of envenoming pertaining to fatal snakebites across PNG, 2018-2023.

Time to treatment	
Time to treatment (hours) Median (IQR)	5.73 (3.00–9.19)
*Data available n (%)*	18 (56.25%)
First aid	
Pressure bandage without immobilisation n (%)	12 (46.15%)
Tourniquet n (%)	5 (19.23%)
None applied n (%)	5 (19.23%)
Data available n (%)	26 (81.25%)
Clinical signs of coagulopathy	
20WBCT n (%)	16 (50.00%)
Bite site bleeding n (%)	12 (37.50%)
Other bleeding n (%)	10 (31.25%)
Haematemesis n (%)	8 (25.00%)
Cuts and abrasions n (%)	7 (21.88%)
Haematuria n (%)	5 (19.23%)
Clinical signs of neurotoxicity	
Ptosis n (%)	30 (93.75%)
Clear speech* n (%)	24 (75.00%)
Swallowing* n (%)	24 (75.00%)
Shrugs shoulders* n (%)	23 (71.88%)
Tongue protrusion* n (%)	22 (68.75%)
Opthalmoplegia n (%)	20 (62.50%)
Mouth open 2 cm* n (%)	19 (59.38%)
Abdominal breathing n (%)	19 (59.38%)
Shortness of breath n (%)	19 (59.38%)
Turns head* n (%)	17 (53.13%)
Patients administered antivenom	
Number of patients n (%)	23 (71.88%)
Vials administered	26

* Normal ability impaired for these measures | Total counts include data from unrecorded patients and values < 5 that have been removed to maintain privacy.

Outcomes of non-fatal snakebites: Outcome data was available for approximately 1 in 5 snakebite patients (n = 539, 20.95%) and 2 in 5 patients administered antivenom during the study period (n = 460, 38.37%; Table 11). For patients administered antivenom, more than 75% were reported to have survived the snakebite or be improving (n = 350, 76.09%). Approximately 1 in 6 patients administered antivenom (n = 77, 16.74%) were recorded as receiving ongoing care, including transfer to other facilities, ICU, intubation, and/or further monitoring. While 33 patients were recorded as having a worsening condition (7.17%).

**TABLE 11:** Non-fatal outcomes for snakebite patients across PNG, 2018-2023.

​	Death adder		Polyvalent		Taipan		Total antivenom		No AV administered		TOTAL	
	*Count*	*%*	*Count*	*%*	*Count*	*%*	*Count*	*%*	*Count*	*%*	*Count*	*%*
Patients administered AV	222	-	442	-	535	-	1,199	-	1,374	-	2,573	-
Data available - outcomes	109	49.10	215	48.64	136	25.42	460	38.37	79	5.69	539	20.95
Survived/Improving	94	86.24	165	76.74	91	66.91	350	76.09	66	83.54	416	77.18
Receiving ongoing care	9	8.26	32	14.88	36	26.47	77	16.74	5	6.33	82	15.21
Patient worsening	6	5.50	18	8.37	9	6.62	33	7.17	8	10.13	41	7.61

No antivenom = total data for patients not administered antivenom | Total antivenom = total data for patients administered antivenom | TOTAL, all patient data.

Rates of patient survival/improvement, rates of patients receiving ongoing care and estimate non-usage rates of antivenom (∼15%) provided by the PNG Partnership team enabled us to estimate survival rates attributable to the work of the Partnership. These rates produced indicative survival rates of between 76.09% and 92.83% for patients administered antivenom. While the bias of these rates is noted due to the missing data, these rates align with both anecdotal reports from the PNG Snakebite Partnership team and key NDoH figures who actively monitor adverse outcomes including fatal bites across Partnership sites and with previously reported case-fatality rates of 10.50% in antivenom poor environments (McGain et al., 2004). These rates also take into account the 15% rate used by the Partnership for antivenom of loss/expiry.

Extrapolating these rates across the snakebite dataset reported here, it is estimated that 901-1,100 patients who received antivenom across PNG during the study period will have survived their snakebites. With >2,700 vials distributed across PNG during this period, the overall estimate may be closer to 1,746-2,130 patients who survived a snakebite following the administration of antivenom between 2018 and 2023. As noted above, these estimates and extrapolations are based on incomplete data. For a more accurate accounting of the impact of this program, further efforts that increase the collection of accurate and complete patient data are required to provide a more finely tuned assessment of both the scale of snakebite and the impact of the Partnership across PNG.

## Discussion

This retrospective study is the first analysis of patient data collected through the PNG Snakebite Partnership. It represents one of the largest epidemiological studies conducted on snakebite in the region to date. While previous studies have focused on snakebite morbidity and mortality in an era of limited antivenom availability and rapidly increasing antivenom costs (McGain et al., 2004; Lalloo et al., 1995a; Lalloo et al., 1995b; Trevett et al., 1995a; Currie et al., 1991; Brian and Vince, 1987; Campbell and Young, 1961; Trevett et al., 1995b; Campbell, 1969), this is the first investigation which reports on snakebite across PNG in an era of freely, and widely, available life-saving antivenoms. This study shows that between 2018 and 2023, 2,573 people were recorded as attending one of the >70 participating health facilities across PNG for the management of a suspected snakebite. 1,199 (46.60%) of these patients were subsequently administered antivenom with an estimated 901–1,100 patients surviving their bites. Extrapolating these findings across the work of the PNG Snakebite Partnership during this time, with >2,700 vials distributed it is estimated that 1,746–2,130 patients survived their bites following administration of antivenom between 2018 and 2023 (assuming a 15% rate of loss/expiry). While promising, as noted above, these estimates and extrapolations are based on incomplete data. For a more accurate accounting of the impact of this program, further efforts that increase the collection of accurate and complete patient data are required to provide a more finely tuned assessment of both the scale of snakebite and the impact of the Partnership across PNG.

Here we report snakebite case rates as high as 462.61 snakebites per 100,000 in some districts (Rigo, Central Province) and 215.10 snakebites per 100,000 in Central Province. These findings closely align with previous reports of incidence rates as high as 526 snakebites per 100,000 at a district level (Kairuku, Central Province) and 215.50 snakebites per 100,000 in Central Province (Lalloo et al., 1995a), reaffirming the considerable public health burden that snakebite envenoming places on communities and health systems across PNG (Lalloo et al., 1995a; Lalloo et al., 1995b; Currie et al., 1991).

Snakebites occurred year-round and impacted people of all ages and sexes. While previous studies have reported antivenom rates as low as 7.2% (Williams et al., 2007), here >46% of patients attending a health centre for a suspected snakebite were administered antivenom; figures consistent with those reported in the Australian healthcare system (S, 2021). The antivenom rates observed should consider that patients attending health facilities in PNG for suspected snakebites will include individuals with injuries sustained from non-snakebite related causes (e.g., standing on a stick in the presence/vicinity of a snake; known as a ‘stick bite’), bites from non-venomous snakes (*i.e.*, pythons, boas, file snakes, *etc.*), and bites from venomous snakes that do not result in clinical envenoming (‘dry bites’). These patients are monitored and treated symptomatically as required, which may include tetanus and antibiotic prophylaxis to avoid infections (Winkel et al., 2005). Patients bitten by venomous snakes who show signs of clinical envenoming are treated with antivenom therapy and monitored until their symptoms are resolved. These patients may also be treated with prophylactic tetanus boosters and antibiotics.

Antivenom administration was broadly appropriate however as noted previously (McGain et al., 2004), a preference for polyvalent antivenom administration was at times shown over the more suitable monovalent alternative. For example, in Central Province, a 20WBCT has a reported positive predictive value of >95% for Papuan taipan envenoming (Trevett et al., 1995a) and it is recommended that envenomed patients with incoagulable blood in Central province should receive monovalent taipan antivenom (McGain et al., 2004). Despite this, 121 patients received polyvalent antivenoms in Central province health facilities after recording a positive 20WBCT. The use of polyvalent in these centres may be attributed to low supplies of monovalent antivenom; however, a more likely explanation is that the polyvalent antivenoms are preferred by some clinicians for their broad-spectrum neutralisation properties, as they do not require the clinical delineation between envenoming syndromes arising from specific snake species. Monovalent taipan antivenom administration would have decreased the risk of adverse reactions for these patients (O'Leary and Isbister, 2009) and would have ensured that polyvalent antivenoms, which are available in fewer quantities, remained available for patients with less certain diagnoses.

Antivenom usage reported here accounts for ∼44% of the antivenom distributed by the Partnership during the study period, leaving ∼1,500 vials unaccounted for. Patient data has reported here from 49/73 (67.12%); however, this data is unlikely to cover the entire study period for each facility. Antivenoms were broadly well-tolerated with adverse reactions recorded at levels consistent with previous reports (Williams et al., 2007). While considerable variability exists in the literature regarding these rates, recent reports of higher rates involve patient populations receiving a median of 4 vials of antivenom (Isbister et al., 2008) compared to the current study where most patients only received a single vial. No relationship was found between the time from snakebite to subsequent treatment and the rate of adverse reactions, despite previous reports of a significant relationship (Williams et al., 2007). The incomplete recording of patient data reported here and the significantly larger sample size of snakebite patient records in the Partnership’s database could both be contributing to this discrepancy. These factors may also be at least partially responsible for the low snakebite fatality rate reported here, and further investigations are required to ensure that the true impact of snakebite is accurately reported in future iterations of this work.

Patients attended health facilities slightly more rapidly in the current study than previously reported, with ∼85% of patients attending a health facility within 9 h post bite (∼77% attending <10 h in (McGain et al., 2004; Trevett et al., 1995a)). However, they remain well above both the median time to antivenom treatment observed in Australia (4 h post bite) and the recommended 2–6 h post bite window to ensure the highest efficacy of the antivenom treatment (Isbister, 2023). To increase the efficacy of antivenom treatment across PNG, efforts must also be made to facilitate earlier access to medical care. The use of appropriate first aid also showed signs of improvement with ∼50% of patients being managed with a pressure bandage with/without immobilisation (compared to ∼30% in (McGain et al., 2004)). However, it is concerning to note that despite efforts to decrease the practise, 1 in 4 patients continue to be managed using potentially dangerous first aid techniques, including tourniquets and/or scarification of the bite site. Tourniquets can cause pain, ischemia, nerve injury, and result in the loss of the affected limb (Parker-Cote and Meggs, 2018), while scarification can increase both the entry of venom to circulatory system, by increasing vascular access to the bite site, and the risk of subsequent infection (*e.g.*, tetanus). These techniques are particularly detrimental for patients bitten by non-venomous snakes or for those received a dry bite (were not envenomed) from a venomous snake. In these cases, the impact of the inappropriate first aid techniques applied were the major medical concern. Further community education is needed to ensure appropriate snakebite first aid is applied, and dangerous techniques avoided.

The reporting of clinical signs of envenoming and the diagnosis of snakebite envenoming in patients subsequently not administered antivenom were consistent with Australian hospitalisation data which indicates that mild envenoming cases may be managed with supporting care (S, 2021). However, these results may also indicate a need for additional training for healthcare workers to ensure the accuracy of clinical care. For example, a positive 20WBCT, which provides evidence of coagulopathy and is reported to be a significant indicator of a patient developing neurotoxicity (Chenhall and Williams, 2005), was reported in 15% of patients not administered antivenom. Factors including remnant detergent, soap, or other dirt/debris in the glass vial, the agitation, mixing or inversion of blood samples during the incubation time, and not waiting the full 20 min to assess coagulation can all lead to false positives (White, 2018; Chenhall and Williams, 2005). While the number of false positives cannot be directly determined, these results highlight the necessity of ongoing training to ensure accurate testing is undertaken at all participating facilities. Similar challenges can be observed with measures of neurotoxicity which require subjective determination of the presence of symptoms in individual patients. However, while the need for ongoing training is apparent, the ability to provide additional training beyond what is already offered remains challenging given the logistical constraints and costs of travel to remote facilities.

The oft-cited challenges of collecting timely and complete epidemiological data in infrastructure poor and logistically challenging environments are again highlighted in the current study where data from only 67.12% of health centres was available for analysis (Abdul-Rahman et al., 2023). The reliance on paper forms for recording snakebite data means that forms must be collected by, or digitally transferred to, the project team for digitisation and analysis. However, logistical constraints preventing or delaying return visits to remote facilities coupled with incomplete data collection by healthcare practitioners means that while the figures outlined in this manuscript provide considerable insights into the impact of snakebite across PNG, they fail to represent its true scale as a public health burden. Efforts are underway to digitise the data collection process itself by incorporating snakebite into the electronic National Health Information System (eNHIS) (Rosewell et al., 2021). Here we recommend that these efforts also focus on increasing the collection and accuracy of follow-up data to account for cases involving prolonged hospitalisations and to better account for fatal snakebites. The digitisation of these reports would enable close-to-real-time data collection and would significantly improve the accuracy of our knowledge concerning the impact that snakebite is having on communities and health systems across the country.

Limitations with the data collected via the patient forms made it difficult to validate the treatments provided against the clinical syndrome of envenoming from specific snake species. Monovalent antivenom usage, 20WBCT results, and our understanding of species distribution across the country were used as surrogates to map out species specific interactions, which, as noted above, indicated that antivenom administration was broadly appropriate. However, given the overlap of clinical symptoms of envenoming caused by medically significant snakes, the lack of availability of venom detection kits, and the incomplete collection/reporting of data via patient forms, definitive clinical validation of the snake species involved was not possible in the current study. Efforts to better capture the snake species involved should be incorporated into future the data collection methodologies.

Missing data across the study variables must also be highlighted as a limitation of the current study. Efforts were made to highlight the extent of data availability throughout the manuscript both to ensure transparency and to highlight the challenges of collecting complete datasets in logistically challenging environments, such as PNG. Missing data can be a significant limitation, and it is not known here whether the data was missing at random or whether specific patients, treatment groups, or regions experienced a greater degree of missing data. It is noted that this data may have introduced some level of bias into the figures reported here.

The success of the PNG Snakebite Partnership remains reliant on governmental support in both PNG and Australia, and on a substantial donation of effective antivenoms from Australian antivenom manufacturer Seqirus, totalling ∼ AUD$8.4 M between 2018 and 2023. The implementation of similar models would therefore be limited to countries with sovereign antivenom manufacturing capacity or the resources to purchase sufficient levels of safe and effective antivenom; a significant challenge to many snakebite-afflicted countries, particularly given current global antivenom production remains insufficient to meet clinical needs (Potet et al., 2021). This means that the ongoing impact of the PNG Snakebite Partnership will be limited by the quantity of vials made available via Seqirus’ donation. Some efforts have been made to increase the availability of effective snakebite medicines for PNG, including the development of a monovalent taipan antivenom by the Instituto Clodomiro Picado (ICP; Costa Rica) (Herrera et al., 2014) and the exploration of the phospholipase A2 inhibitor, varespladib (Gilliam et al., 2023). The ICP taipan antivenom offered a purportedly low-cost antivenom that demonstrated comparable immunoreactivity and neutralising potency to the Seqirus taipan antivenom (Herrera et al., 2014; Vargas et al., 2011). A Phase I/II clinical trial assessing the ICP taipan antivenom against the Seqirus product was completed in 2016 (Australian and New Zealand Clinical, 2012); however, the results of this work are yet to be published. Preclinical investigations of varespladib demonstrated that it was able to reverse neurotoxicity from taipan venom in a porcine model (Gilliam et al., 2023), suggesting that the drug may be an effective adjunct to traditional antivenoms. However, clinical investigations of its efficacy against taipan envenoming have yet to be undertaken. Given their current investigative stages, neither ICP taipan antivenom nor varespladib are likely to supplement existing antivenoms in the short-to-mid-term, and neither offer alternative treatments for patients envenomed by PNG’s other medically significant snake species. Another avenue for securing local production of antivenom would be through the establishment of sovereign antivenom manufacturing in PNG. However, as noted in Ecuador (Ortiz-Prado et al., 2021) and other low-middle income countries (LMICs), the significant costs and technical expertise required to develop local capacity, make such facilities cost-prohibitive and thus unlikely to eventuate.

Like many tropical LMICs, healthcare delivery across PNG remains a challenging proposition. Inadequate infrastructure, ongoing medical supply issues, and limited financial resources, conspire with poor transport, and a large, rural population dispersed across a mountainous landscape, to impede efforts to improve healthcare across the country (World Health Organization, 2024; Schuele and MacDougall, 2022). Despite these challenges, the findings here highlight the capacity to address the public health burden of snakebite even in an environment where physician density remains lower than the LMIC average (PNG: 0.63 doctors per 10,000 people; LMIC average; 7.7 doctors per 10,000 people (Kunjumen, 2021)). The work of the Partnership is in direct alignment with, and offers further support for, the strategic objectives of the World Health Organization’s (WHO’s) snakebite envenoming strategy, with a focus on increasing partnerships, coordination and resources, strengthening health systems, and ensuring access to effective treatments (World Health Organization, 2019).

In conclusion, the first retrospective analysis of patient data collected through the PNG Snakebite Partnership highlights the successful implementation of a medical distribution and training program aligned with the WHO’s strategic objectives. Extrapolated estimates from the data recorded to date indicate that an estimated 901–1,100 patients across PNG have survived snakebites following treatment through the Partnership to date. The data also highlighting the innate challenge of collecting epidemiological data in infrastructure poor environments. Efforts to limit these challenges, if implemented, including adoption of digital data collection methods and further collaboration with provincial health authorities, will continue to increase the resolution of snakebite data in the years to come.

## Data Availability

The data analyzed in this study is subject to the following licenses/restrictions: The patient datasets generated and analysed during the current study were provided by the PNG Snakebite Partnership on behalf of the National Department of Health (PNG). These datasets are not publicly available; however, requests to access this data may be made through the Papua New Guinea Medical Research Advisory Committee. Requests to access these datasets should be directed to https://www.hrp.health.gov.pg/.

## References

[B1] Abdul-Rahman T. Ghosh S. Lukman L. Bamigbade G. B. Oladipo O. V. Amarachi O. R. (2023). Inaccessibility and low maintenance of medical data archive in low-middle income countries: mystery behind public health statistics and measures. J. Infect. Public Health 16 (10), 1556–1561. 10.1016/j.jiph.2023.07.001 37566992

[B2] Australian and New Zealand Clinical Trials Registry, *A Phase I/phase II Randomized Controlled Trial (RCT) of a New Antivenom, Compared to the Currently Used CSL Taipan Antivenom, for the Treatment of the Effects of Papuan Taipan Bite - ACTRN12612001062819* . (2012). p. 1.

[B3] Bal B. Carroll T. Smith R. Williams D. (2005). “Snakebite first aid in Papua New Guinea,” in Venomous Bites and Stings in Papua New Guinea. Editor Williams D. (Australia: Australian Venom Research Unit), 102–118.

[B4] Brian M. J. Vince J. D. (1987). Treatment and outcome of venomous snake bite in children port moresby general hospital, Papua New Guinea. Trans. R. Soc. Trop. Med. Hyg. 81 (5), 850–852. 10.1016/0035-9203(87)90051-4 3450009

[B5] Brown S. G. (2004). Clinical features and severity grading of anaphylaxis. J. Allergy Clin. Immunol. 114 (2), 371–376. 10.1016/j.jaci.2004.04.029 15316518

[B6] Campbell C. H. (1969). Clinical aspects of snake bite in the Pacific area. Toxicon 7 (1), 25–28. 10.1016/0041-0101(69)90158-5 5804762

[B7] Campbell C. H. Young L. N. (1961). The symptomatology, clinical course and successful treatment of papuan elapine snake envenomation. Med. J. Aust. 48 (1), 478–486. 10.5694/j.1326-5377.1961.tb24234.x 13690210

[B8] Chenhall A. Williams D. (2005). “The assessment and treatment of coagulopathy,” in Venomous Bites and Stings in Papua New Guinea. Editor Williams D. (Melbourne, Australia: Australian Venom Research Unit).

[B9] Currie B. J. (2000). Snakebite in tropical Australia, Papua New Guinea and irian jaya. Emerg. Med. 12 (4), 285–294. 10.1046/j.1442-2026.2000.00150.x

[B10] Currie B. J. Sutherland S. K. Hudson B. J. Smith A. M. (1991). An epidemiological study of snake bite envenomation in Papua New Guinea. Med. J. Aust. 154 (4), 266–268. 10.5694/j.1326-5377.1991.tb121088.x 1994203

[B11] Gilliam L. L. Gilliam J. Samuel S. P. Carter R. W. Ritchey J. Bulfone T. (2023). Oral and IV varespladib rescue experiments in juvenile pigs with weakness induced by Australian and papuan Oxyuranus scutellatus venoms. Toxins (Basel) 15 (9). 10.3390/toxins15090557 37755983 PMC10537020

[B12] Herrera M. Paiva O. K. Pagotto A. H. Segura A. Serrano S. M. T. Vargas M. (2014). Antivenomic characterization of two antivenoms against the venom of the taipan, Oxyuranus scutellatus, from Papua New Guinea and Australia. Am. J. Trop. Med. Hyg. 91 (5), 887–894. 10.4269/ajtmh.14-0333 25157124 PMC4228881

[B13] Isbister G. K. (2006). Snake bite: a current approach to management. Aust. Prescr. 29, 125–129. 10.18773/austprescr.2006.078

[B14] Isbister G. K. (2023). Antivenom availability, delays and use in Australia. Toxicon 17, 100145. 10.1016/j.toxcx.2022.100145 36523639 PMC9747507

[B15] Isbister G. K. Brown S. G. MacDonald E. White J. Currie B. J. (2008). Current use of Australian snake antivenoms and frequency of immediate-type hypersensitivity reactions and anaphylaxis. Med. J. Aust. 188 (8), 473–476. 10.5694/j.1326-5377.2008.tb01721.x 18429716

[B16] Jensen S. (2005). “Symptoms and signs of snakebite in Papua New Guinea,” in Venomous Bites and Stings in Papua New Guinea. Editor Williams D. (Australia: Australian Venom Research Unit), 86–101.

[B17] Johnston C. I. Ryan N. M. O'Leary M. A. Brown S. G. A. Isbister G. K. (2017). Australian taipan (oxyuranus spp.) envenoming: clinical effects and potential benefits of early antivenom Therapy - australian snakebite project (ASP-25). Clin. Toxicol. (Phila) 55 (2), 115–122. 10.1080/15563650.2016.1250903 27903075

[B18] Kunjumen T. (2021). Health workers density (per 10 000 population), by occupation. Available online at: https://www.who.int/data/gho/data/indicators/indicator-details/GHO/medical-doctors-(per-10-000-population (Accessed October 9, 2024).

[B19] Kuruppu S. Smith A. I. Isbister G. K. Hodgson W. C. (2008). Neurotoxins from australo-papuan elapids: a biochemical and pharmacological perspective. Crit. Rev. Toxicol. 38 (1), 73–86. 10.1080/10408440701703964 18161503

[B20] Lalloo D. G. Trevett A. J. Saweri A. Naraqi S. Theakston R. D. Warrell D. A. (1995a). The epidemiology of snake bite in central province and national capital district, Papua New Guinea. Trans. R. Soc. Trop. Med. Hyg. 89 (2), 178–182. 10.1016/0035-9203(95)90485-9 7778143

[B21] Lalloo D. G. Trevett A. J. Korinhona A. Nwokolo N. Laurenson I. F. Paul M. (1995b). Snake bites by the papuan taipan (Oxyuranus scutellatus canni): paralysis, hemostatic and electrocardiographic abnormalities, and effects of antivenom. Am. J. Trop. Med. Hyg. 52 (6), 525–531. 10.4269/ajtmh.1995.52.525 7611559

[B22] Lalloo D. G. Trevett A. J. Black J. Mapao J. Saweri A. Naraqi S. (1996). Neurotoxicity, anticoagulant activity and evidence of rhabdomyolysis in patients bitten by death adders (acanthophis sp.) in southern Papua New Guinea. Qjm 89 (1), 25–35. 10.1093/oxfordjournals.qjmed.a030134 8730340

[B23] McGain F. Limbo A. Williams D. J. Didei G. Winkel K. D. (2004). Snakebite mortality at port moresby general hospital, Papua New Guinea, 1992-2001. Med. J. Aust. 181 (11-12), 687–691. 10.5694/j.1326-5377.2004.tb06525.x 15588214

[B24] O'Leary M. A. Isbister G. K. (2009). Commercial monovalent antivenoms in Australia are polyvalent. Toxicon 54 (2), 192–195. 10.1016/j.toxicon.2009.04.004 19375442

[B25] Ortiz-Prado E. Yeager J. Andrade F. Schiavi-Guzman C. Abedrabbo-Figueroa P. Terán E. (2021). Snake antivenom production in Ecuador: poor implementation, and an unplanned cessation leads to a call for a Renaissance. Toxicon 202, 90–97. 10.1016/j.toxicon.2021.09.014 34571098

[B26] Paiva O. Pla D. Wright C. E. Beutler M. Sanz L. Gutiérrez J. M. (2014). Combined venom gland cDNA sequencing and venomics of the new Guinea small-eyed snake, Micropechis ikaheka. J. Proteomics 110, 209–229. 10.1016/j.jprot.2014.07.019 25109465

[B27] Pameh W. (2016). Adolescent health in Papua New Guinea: time for action. P. N. G. Med. J. 59 (1-2), 20–22.

[B28] Parker-Cote J. Meggs W. J. (2018). First aid and pre-hospital management of venomous snakebites. Trop. Med. Infect. Dis. 3 (2). 10.3390/tropicalmed3020045 30274441 PMC6073535

[B29] Pla D. Paiva O. K. Sanz L. Beutler M. Wright C. E. Calvete J. J. (2014). Preclinical efficacy of Australian antivenoms against the venom of the small-eyed snake, Micropechis ikaheka, from Papua New Guinea: an antivenomics and neutralization study. J. Proteomics 110, 198–208. 10.1016/j.jprot.2014.06.016 24980637

[B30] Potet J. Beran D. Ray N. Alcoba G. Habib A. G. Iliyasu G. (2021). Access to antivenoms in the developing world: a multidisciplinary analysis. Toxicon X 12, 100086. 10.1016/j.toxcx.2021.100086 34786555 PMC8578041

[B31] Rosewell A. Shearman P. Ramamurthy S. Akers R. (2021). Transforming the health information system using mobile and geographic information technologies, Papua New Guinea. Bull. World Health Organ 99 (5), 381–387a. 10.2471/BLT.20.267823 33958826 PMC8061671

[B32] S P. (2021). “Venomous bites and stings, 2017–18,” in Injury Research and Statistics (Canberra, Australia: Australian Institute of Health and Welfare).

[B33] Schuele E. MacDougall C. (2022). The missing bit in the middle: implementation of the nationals health services standards for Papua New Guinea. PLoS One 17 (6), e0266931. 10.1371/journal.pone.0266931 35749442 PMC9231790

[B34] Sitprija V. (2006). Snakebite nephropathy (review article). Nephrology 11 (5), 442–448. 10.1111/j.1440-1797.2006.00599.x 17014559

[B35] Trevett A. J. Lalloo D. G. Nwokolo N. C. Naraqi S. Kevau I. H. Theakston R. D. (1995a). The efficacy of antivenom in the treatment of bites by the papuan taipan (Oxyuranus scutellatus canni). Trans. R. Soc. Trop. Med. Hyg. 89 (3), 322–325. 10.1016/0035-9203(95)90562-6 7660450

[B36] Trevett A. J. Lalloo D. G. Nwokolo N. C. Theakston D. G. Naraqi S. Warrell D. A. (1995b). Venom detection kits in the management of snakebite in central province, Papua New Guinea. Toxicon 33 (5), 703–705. 10.1016/0041-0101(94)00179-c 7660375

[B37] Vargas M. Segura A. Herrera M. Villalta M. Estrada R. Cerdas M. (2011). Preclinical evaluation of caprylic acid-fractionated IgG antivenom for the treatment of taipan (Oxyuranus scutellatus) envenoming in Papua New Guinea. PLoS Negl. Trop. Dis. 5 (5), e1144. 10.1371/journal.pntd.0001144 21610854 PMC3096592

[B38] Warrell D. A. Hudson B. J. Lalloo D. G. Trevett A. J. Whitehead P. Bamler P. R. (1996). The emerging syndrome of envenoming by the new Guinea small-eyed snake micropechis ikaheka. QJM An Int. J. Med. 89 (7), 523–530. 10.1093/qjmed/89.7.523 8759493

[B39] White J. (2018). Snakebite & Spiderbite: Management Guidelines South Australia. Australia.

[B40] White J. Mahmood M. A. Alfred S. Thwin K. T. Kyaw K. M. Zaw A. (2019). A comprehensive approach to managing a neglected, neglected tropical disease; the myanmar snakebite project (MSP). Toxicon 1, 100001. 10.1016/j.toxcx.2018.100001 32831344 PMC7285917

[B41] Williams D. (2005). “Snakebite in Papua New Guinea,” in Venomous Bites and Stings in Papua New Guinea. Editor Williams D. (Australia: Australian Venom Research Unit), 5–32.

[B42] Williams D. Wüster W. (2005). “Snakes of Papua New Guinea,” in Venomous Bites and Stings in Papua New Guinea. Editor Williams D. (Melbourne, Australia: Australian Venom Research Unit).

[B43] Williams D. J. Jensen S. D. Nimorakiotakis B. Müller R. Winkel K. D. (2007). Antivenom use, premedication and early adverse reactions in the management of snake bites in rural Papua New Guinea. Toxicon 49 (6), 780–792. 10.1016/j.toxicon.2006.11.026 17210167

[B44] Winkel K. D. McGain F. Nimorakiotakis B. Williams D. (2005). “The role and use of antivenom in Papua New Guinea,” in Venomous Bites and Stings in Papua New Guinea. Editor Williams D. (Melbourne, Australia: Australian Venom Research Unit).

[B45] World Health Organization (2019). Snakebite Envenoming: A Strategy for Prevention and Control. Geneva, Switzerland: World Health Organization.

[B46] World Health Organization (2024). Papua New Guinea–WHO Country Cooperation Strategy 2024–2028. Papua New Guinea: World Health Organization.

[B47] Wüster W. Dumbrell A. J. Hay C. Pook C. E. Williams D. J. Fry B. G. (2005). Snakes across the strait: trans-torresian phylogeographic relationships in three genera of Australasian snakes (serpentes: elapidae: acanthophis, oxyuranus, and pseudechis). Mol. Phylogenetics Evol. 34 (1), 1–14. 10.1016/j.ympev.2004.08.018 15579378

