# Supplementary material for: The Papua New Guinea snakebite partnership: a retrospective study of snakebite epidemiology and management, 2018-2023
Source: Front Toxicol. 2026 Oct 12;8:1961869. doi: 10.3389/ftox.2026.1961869 (PMC13719242; doi:10.3389/ftox.2026.1961869)
Supplement: Supplementary file 1 [file Supplementaryfile1.docx]

**Supplementary material for The Papua New Guinea Snakebite Partnership: A retrospective study of snakebite epidemiology and management, 2018-2023**

Geno Roalakona, Andrew Maru, Timothy N. W. Jackson, Teatulohi Matainaho, Garry Nou, Nakapi Tefuarani, Andrew D Watt

[SUPPLEMENTARY RESULTS	1](#_Toc199756487)

[Bites by time of day and year	1](#_Toc199756488)

[SUPPLEMENTARY TABLES AND FIGURES	2](#_Toc199756489)

# SUPPLEMENTARY RESULTS

## Bites by time of day and year

Snakebites occurred at all times of the day with the highest number occurring between 18:00-20:59 (***Supplementary Table 2***; n = 642, 25.57%). The lowest number of bites were recorded in the early morning, with only 1.84% of bites occurring before 06:00. High bite numbers coincided with time periods associated with higher levels of work, activity, and movement of peoples with over 420 recorded each 3-hour period between 09:00 - 20:59. Taipan bites were more prevalent in the middle of the day, with a third of bites occurring between 12:00 – 14:59 (n = 171). While death adder bites were highest between 15:00 – 17:59 (n = 58, 26.85%). Time of bite data was available for 2,506 patients (97.40%).

## Description of snake

A total of 1,621 patients (63.00%) reported seeing the snake around the time of the bite. Of these, 1,465 (90.37%) were able to provide a description (***Supplementary*** ***Table 3***). More than 45% of patients gave a description of a snake consistent with a Papuan Taipan (*Oxyuranus scutellatus*; n = 688, 46.96%; descriptions included black snake/long black snake, black snake red back, black snake white belly, Papuan black, Taipan). While *Papuan black* could also describe the rare Papuan blacksnake (*Pseudechis papuanus*), it is a local term which is predominately used to describe taipans. Only 22 patients described the taipan by name (1.50%) while 100 patients (6.83%) named the death adder (*Acanthophis laevis* or *Acanthophis rugosus;* a further 310 described seeing a ‘short snake’, 21.16%), and 11 patients (0.75%) noted seeing a small-eyed snake (*Micropechis ikaheca*). Other descriptions were provided by 221 patients (15.09%).

**Activity undertaken at time of bite**

More than half of snakebites occurred when people were walking (n = 1,133, 51.52%; ***Supplementary Table 4***), including individuals who were just walking (n = 599, 27.24%), walking on a bush track (n = 270, 12.28%), walking home (n = 150, 6.82%), or walking in other areas including the road, river, beach, village, market, swamp, and/or mangrove (n = 114, 5.18%). Gardening was the next most common activity being undertaken when a snakebite occurred, with just over one in five patients being bitten while gardening (n = 467, 21.24%). The remaining activities (*e.g.*, Hunting / fishing, Collecting fruit, vegetables, betelnut, or other plant materials, Other, Working, Washing / Bathing / Fetching water, Playing, Around the toilet, Collecting firewood / making a fire, Resting / Relaxing / sleeping, Around the home / yard) accounted for just over a quarter of patients (n = 599, 27.24%) with hunting / fishing the only activity accounting for >5% of snakebites (n = 121, 5.50%).

Data regarding the activity that individuals were undertaking when a snakebite occurred was available for 2,199/2,573 (85.46%) patients. Activities listed also include the journey to and from the activity (*e.g.*, gardening included the act of gardening in addition to the individual walking to or back from the garden). Walking home was an exception to this rule and was instead listed as a sub-category of walking.

# SUPPLEMENTARY TABLES AND FIGURES

***Supplementary Table 1. Snakebite patients attended health facilities across 11 provinces in PNG between 2018-2023.*** *Data aggregated by sex and age.*

|  | **Female** | | **Male** | | **Adult** | | **Child** | | **Total** | |
| --- | --- | --- | --- | --- | --- | --- | --- | --- | --- | --- |
|  | **Count** | **%** | **Count** | **%** | **Count** | **%** | **Count** | **%** | **Count** | **%** |
| **Central** | 582 | 71.32 | 969 | 74.08 | 1,120 | 73.44 | 396 | 72.13 | 1,574 | 72.80 |
| **East Sepik** | 42 | 5.15 | 61 | 4.66 | 64 | 4.20 | 37 | 6.74 | 103 | 4.76 |
| **Eastern Highlands** | - | - | 26 | 1.99 | 19 | 1.25 | 14 | 2.55 | 34 | 1.57 |
| **Gulf** | 37 | 4.53 | 51 | 3.90 | 68 | 4.46 | 15 | 2.73 | 88 | 4.07 |
| **Madang** | 20 | 2.45 | 27 | 2.06 | 35 | 2.30 | 11 | 2.00 | 49 | 2.27 |
| **Milne Bay** | 20 | 2.45 | 43 | 3.29 | 42 | 2.75 | 20 | 3.64 | 63 | 2.91 |
| **Morobe** | 19 | 2.33 | 17 | 1.30 | 30 | 1.97 | - | - | 37 | 1.71 |
| **Oro** | 31 | 3.80 | 45 | 3.44 | 57 | 3.74 | 13 | 2.37 | 76 | 3.52 |
| **Western** | 65 | 7.97 | 68 | 5.20 | 90 | 5.90 | 43 | 7.83 | 135 | 6.24 |
| **Patients with provincial data** | 816 | 100.00 | 1,308 | 100.00 | 1,525 | 100.00 | 549 | 100.00 | 2,162 | 100.00 |
| *Note: Total counts include data from unrecorded patients and values <10 that have been removed to maintain privacy* | | | | | |  |  |  |  |  |

***Time Bitten***

***Supplementary Table 2. Time of bite data for snakebite patients based on antivenom administration across PNG, 2018-2023***

|  | **Death Adder** | | **Polyvalent** | | **Taipan** | | **Total Antivenom** | | **No Antivenom** | | **TOTAL** | |
| --- | --- | --- | --- | --- | --- | --- | --- | --- | --- | --- | --- | --- |
|  | Count | % | Count | % | Count | % | Count | % | Count | % | Count | % |
| **00:00 - 02:59** | - | - | - | - | - | - | 7 | 0.60% | 9 | 0.67% | 16 | 0.64% |
| **03:00 - 05:59** | - | - | - | - | - | - | 9 | 0.78% | 21 | 1.56% | 30 | 1.20% |
| **06:00 - 08:59** | 16 | 7.41% | 34 | 7.93% | 44 | 8.53% | 94 | 8.10% | 99 | 7.36% | 193 | 7.70% |
| **09:00 - 11:59** | 33 | 15.28% | 71 | 16.55% | 136 | 26.36% | 240 | 20.67% | 178 | 13.23% | 418 | 16.68% |
| **12:00 - 14:59** | 49 | 22.69% | 109 | 25.41% | 171 | 33.14% | 329 | 28.34% | 229 | 17.03% | 558 | 22.27% |
| **15:00 - 17:59** | 58 | 26.85% | 111 | 25.87% | 112 | 21.71% | 281 | 24.20% | 233 | 17.32% | 514 | 20.51% |
| **18:00 - 20:59** | 49 | 22.69% | 88 | 20.51% | 39 | 7.56% | 176 | 15.16% | 466 | 34.65% | 642 | 25.62% |
| **21:00 - 23:59** | 6 | 2.78% | 11 | 2.56% | 8 | 1.55% | 25 | 2.15% | 110 | 8.18% | 135 | 5.39% |
| **Patients with time data** | 216 | 100.00% | 429 | 100.00% | 516 | 100.00% | 1,161 | 100.00% | 1,345 | 100.00% | 2,506 | 100.00% |
| *Note: Total counts include data from unrecorded patients and values <5 that have been removed to maintain privacy* | | | | | | | | | | |  |  |
| *No antivenom = total data for patients not administered antivenom \| Total antivenom = total data for patients administered antivenom \| TOTAL = All patient data* | | | | | | | | | | | | |

***Description of snake***

***Supplementary Table 3. Overview of descriptions that patients provided of the snake seen around the time of the snakebite***

| **Description** | **Count** | **%** |
| --- | --- | --- |
| Black snake or long black snake | 484 | 33.04 |
| Short Snake | 310 | 21.16 |
| Other snake description | 221 | 15.09 |
| Death adder | 100 | 6.83 |
| Brown snake | 90 | 6.14 |
| Black snake red back | 77 | 5.26 |
| Black snake white belly | 56 | 3.82 |
| Papuan Black | 49 | 3.34 |
| Taipan | 22 | 1.50 |
| Black / brown snake | 20 | 1.37 |
| Yellow snake | 18 | 1.23 |
| Small eyed snake | 11 | 0.75 |
| Big snake (unknown colour) | 7 | 0.48 |
| **Patients with snake description data** | **1,465** | **100.00** |

***Supplementary Table 4. Overview of activity data when snakebite occurred by sex and age for snakebites occurring across PNG, 2018-2023***

|  | **Death Adder** | | **Polyvalent** | | **Taipan** | | **Total Antivenom** | | **TOTAL** | |
| --- | --- | --- | --- | --- | --- | --- | --- | --- | --- | --- |
|  | Count | % | Count | % | Count | % | Count | % | Count | % |
| **Walking** | 86 | 45.74 | 191 | 49.23 | 236 | 52.91 | 513 | 50.20 | 1,133 | 51.52 |
| *- Walking* | 49 | 26.06 | 91 | 23.45 | 126 | 28.25 | 266 | 26.03 | 599 | 27.24 |
| *- Walking on a bush track* | 14 | 7.45 | 57 | 14.69 | 78 | 17.49 | 149 | 14.58 | 270 | 12.28 |
| *- Walking home* | 12 | 6.38 | 24 | 6.19 | 15 | 3.36 | 51 | 4.99 | 150 | 6.82 |
| *- Other walking (road, river, beach, village, market, swamp, mangrove, etc)* | 11 | 5.85 | 19 | 4.90 | 17 | 3.81 | 47 | 4.60 | 114 | 5.18 |
| **Gardening** | 50 | 26.60 | 97 | 25.00 | 91 | 20.40 | 238 | 23.29 | 467 | 21.23 |
| **Hunting / fishing** | 15 | 7.98 | 26 | 6.70 | 26 | 5.83 | 67 | 6.56 | 121 | 5.50 |
| **Collecting fruit, vegetables, betelnut, or other plant materials** | 10 | 5.32 | 22 | 5.67 | 21 | 4.71 | 53 | 5.19 | 96 | 4.37 |
| **Other** | **-** | - | - | - | - | - | 22 | 2.15 | 64 | 2.91 |
| **Working** | **-** | - | - | - | - | - | 21 | 2.05 | 55 | 2.50 |
| **Washing / Bathing / Fetching water** | 7 | 3.72 | 5 | 1.29 | 5 | 1.12 | 17 | 1.66 | 50 | 2.27 |
| **Playing** | 8 | 4.26 | 8 | 2.06 | 10 | 2.24 | 26 | 2.54 | 48 | 2.18 |
| **Around the toilet** | **-** | - | - | - | - | - | 16 | 1.57 | 47 | 2.14 |
| **Collecting firewood / making a fire** | **-** | - | - | - | - | - | 27 | 2.64 | 47 | 2.14 |
| **Resting / Relaxing / sleeping** | **-** | - | **-** | - | - | - | 14 | 1.37 | 37 | 1.68 |
| **Around the home / yard** | **-** | - | **-** | - | - | - | 8 | 0.78 | 34 | 1.55 |
| **Patients with activity data** | 188 | 84.68 | 388 | 87.78 | 446 | 83.36 | 1,022 | 85.24 | 2,199 | 85.46 |
| **Total patients** | 222 |  | 442 |  | 535 |  | 1,199 |  | 2,573 |  |
| *Note: Total counts include data from unrecorded patients and values <5 that have been removed to maintain privacy. \| Other includes cleaning/clearing rubbish, cooking, interacting with the snake and exercising (swimming, running, riding a bike). \| Total antivenom = total data for patients administered antivenom \| TOTAL = All snakebite patient data. \| % of activity data based on number of patients with activity data \| % of 'Patients with activity data' based on Total patients.* | | | | | | | | | | |

***Premedications and adverse reactions to AV***

***Supplementary Table 5. Premedications administered to patients administered antivenom across PNG, 2018-2023***

|  | **Death Adder** | | **Polyvalent** | | **Taipan** | | **Total** | |
| --- | --- | --- | --- | --- | --- | --- | --- | --- |
|  | **Count** | **%** | **Count** | **%** | **Count** | **%** | **Count** | **%** |
| **Adrenaline** | 133 | 59.91 | 252 | 57.01 | 388 | 72.52 | 773 | 64.47 |
| **Hydrocortisone** | 27 | 12.16 | 88 | 19.91 | 30 | 5.61 | 145 | 12.09 |
| **Other** | - | - | - | - | - | - | 5 | 0.42 |
| **Patients receiving premedication** | 162 | 72.97 | 342 | 77.38 | 419 | 78.32 | 923 | 76.98 |
| **Vials administered** | 222 |  | 442 |  | 535 |  | 1,199 |  |
